# Involvement of Right STS in Audio-Visual Integration for Affective Speech Demonstrated Using MEG

**DOI:** 10.1371/journal.pone.0070648

**Published:** 2013-08-12

**Authors:** Cindy C. Hagan, Will Woods, Sam Johnson, Gary G. R. Green, Andrew W. Young

**Affiliations:** 1 Department of Psychology, University of York, Heslington, York, United Kingdom; 2 York Neuroimaging Centre, University of York, Heslington, York, United Kingdom; 3 Brain Mapping Unit, Department of Psychiatry, University of Cambridge, Cambridge, United Kingdom; 4 Brain and Psychological Sciences Research Centre, Swinburne University of Technology, Hawthorn, Melbourne, Victoria, Australia; CEA.DSV.I2BM.NeuroSpin, France

## Abstract

Speech and emotion perception are dynamic processes in which it may be optimal to integrate synchronous signals emitted from different sources. Studies of audio-visual (AV) perception of neutrally expressed speech demonstrate supra-additive (i.e., where AV>[unimodal auditory+unimodal visual]) responses in left STS to crossmodal speech stimuli. However, emotions are often conveyed simultaneously with speech; through the voice in the form of speech prosody and through the face in the form of facial expression. Previous studies of AV nonverbal emotion integration showed a role for right (rather than left) STS. The current study therefore examined whether the integration of facial and prosodic signals of emotional speech is associated with supra-additive responses in left (cf. results for speech integration) or right (due to emotional content) STS. As emotional displays are sometimes difficult to interpret, we also examined whether supra-additive responses were affected by emotional incongruence (i.e., ambiguity). Using magnetoencephalography, we continuously recorded eighteen participants as they viewed and heard AV congruent emotional and AV incongruent emotional speech stimuli. Significant supra-additive responses were observed in right STS within the first 250 ms for emotionally incongruent and emotionally congruent AV speech stimuli, which further underscores the role of right STS in processing crossmodal emotive signals.

## Introduction

Emotional displays can often be both seen and heard, whether they occur as the temper tantrum of a toddler or as the outcries against a foul called on your favorite football team. Moreover, emotion can be expressed either verbally (the cry of “foul!” or expletive when the referee seems to have looked the wrong way) or nonverbally (the gasp when you observe a narrowly missed goal by your favorite striker).

Despite this common observation, a widespread tendency in emotion research is to treat faces and voices as independent, parallel signals. Yet studies have shown that facial and vocal cues may interact in such a way that multisensory integration can facilitate the rapid detection and identification of another's emotional disposition [1], [2]. With respect to emotive facial and vocal signals this is a particularly strong possibility since crossmodal integration is already known to occur in speech perception [3]. The debate can be seen in that while one well-respected model of face perception suggests that the superior temporal sulcus (STS) processes facial expressions and other transient facial signals [4], [5], other researchers [6] note that emotional displays are often audio-visual (AV) by nature and suggest the STS is an integrating region aiding in the overall perception of emotion by ear and by eye.

If the STS is involved in the audio-visual integration of emotion, one might expect that STS possesses mixed neuronal populations (auditory, visual, and audio-visual) and responds quickly to AV emotion given the ecological importance of perceiving emotional displays. An important consideration is the neuroimaging metric used to identify sites of crossmodal integration, with Calvert and colleagues [7] highlighting that while conjunction (AV>A∩AV>V), intersection (A∩V) and interaction (i.e., supra-additivity) analyses all identify STS as a site for human speech integration, interaction techniques confer the additional benefit of mirroring the electrophysiological properties of neuronal cells involved in signal integration [8], [9], and hence possibly offer additional sensitivity for crossmodal region identification [7], [10], [11]. It is noteworthy that conjunction and intersection analyses identify bilateral STS responses whereas interaction techniques identify more circumscribed unilateral responses in left STS during the integration of neutrally expressed human speech [7]. Although in different hemispheres, the unilateral STS response is in line with our magnetoencephalography (MEG) demonstration of supra-additive responses in right STS within the first 250 ms when participants were presented with a facial+vocal nonverbal fear stimulus [12].

The contrast between a predominantly right-sided STS response to audio-visual nonverbal signals of emotion and a left-sided STS response for audio-visual speech [7], [12], [13], poses an intriguing question: which brain region is more involved in integrating facial and vocal signals of affective speech? Based on the observations made by Calvert and colleagues [7], [13] it would appear that left STS is involved in audio-visual integration that underpins the comprehension of speech content. For speech with emotional as well as semantic content, however, matters are more complex. “Tone of voice” is an important tool used to convey feelings. This speech prosody, or the natural inflection and rhythm of emotional speech, occurs alongside dynamic facial signals of emotion in everyday conversation. Interestingly, patients with brain injury to the right hemisphere display impairments in interpreting and identifying a range of nonverbal signals, including those depicted in both photographs of different facial expressions and sentences spoken with emotional prosody (i.e., *how* a word is spoken; [14]). A more recent study using dynamic causal modeling to infer the relative timing of inputs to brain regions pre-specified by the various models under scrutiny, observed a temporal advantage for the right temporal lobe in processing the acoustic properties of a prosodic stimulus relative to further higher-order emotional processing taking place in the frontal lobe [15].

The position becomes less clear in the context of existing neuroimaging studies of emotional speech integration where bilateral STS responses have been reported [16], [17]. However, these two studies performed conjunction analyses, which may partly explain the bilateral effects observed. Whether bilateral of unilateral STS responses would be observed with an interaction analysis of emotional speech is unknown. To address this question, we created audio-visual stimuli with neutral semantic content (non-emotional words) spoken with emotionally prosodic and facially expressive gestures. By using neutral words, we ensured that any responses to perceived emotion came from the non-verbal facial or prosodic cues, allowing us to investigate neural mechanisms involved in audio-visual interpretation of expressed emotion without the contaminating effects of semantic speech content. While a previous study used MEG to examine areas of cortex displaying greater activity for bimodal over unimodal emotion conditions [18], we report the first MEG study to examine the location and neural time course of supra-additive multisensory integration for facial and vocal signals of affective speech. Building on techniques developed for our previous study of audio-visual integration of nonverbally expressed emotion [12], MEG was used to provide information about the neural time course, frequency content, and location of crossmodal emotion integration.

A further question of interest was whether emotional ambiguity arising through incongruence between information carried in visual and auditory channels modulates any supra-additive brain responses observed in STS. While Calvert et al. [13] reported that incongruent AV speech stimuli elicited sub-additive responses in STS when compared to congruent AV speech, other studies have failed to replicate this finding [19], [20]. Dynamic facial and vocal signals of fear and disgust were used as exemplars of emotion and were presented unimodally (face only or voice only) and across visual plus auditory sensory modalities. We chose the emotions fear and disgust because the neuropsychological literature strongly implicates the neural regions underlying these emotions as sensitive to both auditory and visual emotive signals [21]–[24]. The visual and auditory displays were either incongruent (i.e., fear prosody+disgust facial expression; disgust prosody+fear facial expression) or congruent (i.e., fear prosody+fear facial expression; disgust prosody+disgust facial expression) for emotional expression across sensory streams. Participants were required only to passively monitor the stimuli while performing an irrelevant task (looking for a small letter); there was no requirement to respond to the emotional or to the semantic content of the presented speech stimulus.

To examine crossmodal responses during emotion integration, we used a strict criterion of supra-additive power derived from our previous study of nonverbal emotion [12]. Following from our previous work and lesion and fMRI studies of affective prosody [12], [14], [25], our hypotheses were that the integration of emotional cues demonstrated through dynamic facial expression and vocal prosody occurs in the right STS (or nearby regions) and that integration will occur quickly, within the first 250 ms of stimulus presentation [1], [12], [26], [27].

## Materials and Methods

### Participants

Twenty healthy participants from the University of York were recruited to take part in the study and provided written informed consent. Two participants were excluded due to excessive artefact in the MEG data (discussed further in *Analysis of Imaging Data* subsection), leaving a total of eighteen participants (8 male; mean age ± SD = 23.62±3.46, range = 18.88–30.98 years). One of these participants was also part of a separate group who validated the stimuli in a previous experiment and therefore was exposed to a fraction of the stimuli months prior to the current neuroimaging experiment. All participants were without history of neurological injury and were right-handed, with normal hearing, and normal or corrected-to-normal vision. Participants were offered a small stipend for study participation. The study was jointly approved by the ethics committees of the University of York, Department of Psychology and University of York, York Neuroimaging Centre and is in accord with the Declaration of Helsinki.

### Experimental Stimuli

#### Video Stimuli Creation and Validation

AV stimuli capturing the head and shoulders of seven actors were recorded with a digital video camera. Twenty monosyllabic words with low scores in both valence and arousal (i.e., affectively neutral) were selected from the Affective Norms for English Words (ANEW; [28]) to be spoken by each actor with fearful or disgusted affective expression and intonation. Unimodal visual, unimodal auditory and AV stimuli were created by utilizing either the visual, auditory (paired with a visual track of a solid grey screen background) or both portions of the film clip. Each stimulus was rated for intensity of fear and disgust (order counterbalanced) over the course of two consecutive days by nine raters and were expected to meet two criteria: each stimulus had to be highly recognizable as the intended emotion; and each stimulus had to be highly discernible from the unintended emotion. Mean rating responses were used to identify the best-rated and most distinctive video depictions of fear and disgust emotional expressions. Ten ANEW words (Table S1 in Information S1) spoken by three male actors (DM, MH & GL) were identified as stimuli suitable for use in subsequent experiments, yet actors DM and GL were selected for use in the neuroimaging experiment described below as four independent raters rated these two actors as being the most distinct from each other in both visual and auditory domains. See Information S1 for a more detailed description of the stimulus ratings and validation procedure (Tables S2, S3, S4 in Information S1).

#### Visual stimuli

**Figure 1:**
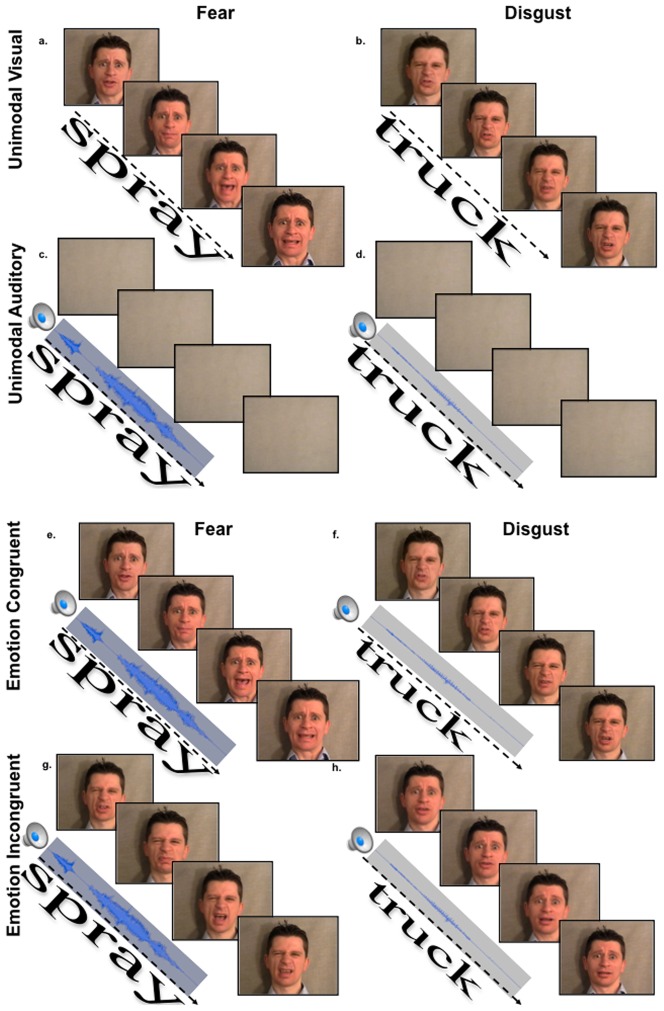
Depictions of the unimodal and bimodal stimulus types. Stimulus representations of (a–b) unimodal visual emotion, and (c–d) unimodal auditory emotion conditions are shown. The unimodal visual emotion condition was comprised of (a) unimodal visual fear, and (b) unimodal visual disgust stimuli. The unimodal auditory emotion condition was comprised of (c) unimodal auditory fear, and (d) unimodal auditory disgust stimuli. Stimulus representations of (e–f) audio-visual congruent emotion, (e) congruent fear, (f) congruent disgust, and (g–h) incongruent emotion conditions are shown. The audio-visual congruent emotion condition was comprised of (e) congruent fear and (f) congruent disgust stimuli. Audio-visual (g–h) incongruent emotion stimuli were created by swapping the visual tracks across fear and disgust stimuli, while keeping the word spoken across auditory and visual tracks consistent.

Video clips (the visual component of a video recording) of two non-professional male actors (DM, GL) speaking neutral words with fearful and disgusted facial expressions were used. The faces were presented upon a grey background to minimize contrast differences between stimuli (**Figure 1a–b**).

A total of forty expressions were selected based upon high recognition ratings for fearful and disgusted expressions (detail about the selection procedure can be found in Information S1). Each facial expression was repeated three times to generate a total of 120 visual stimulus presentations.

#### Auditory stimuli

Sound clips (the auditory component of the video recording) of two non-professional male actors (DM, GL) speaking neutral words with fearful (**Figure 1c**) and disgusted (**Figure 1d**) vocal intonations were used. Forty affective vocal intonations (20 fear and 20 disgust) were included based upon high recognition ratings for fearful and disgusted intonations (see Information S1). Each vocal intonation was repeated three times for a total of 120 auditory stimulus presentations.

#### Audio-Visual stimuli

Each face was paired with a fearful or disgusted vocal intonation of the corresponding neutral word to make both congruent emotion (i.e., unambiguous) (**Figure 1a–b**) and incongruent emotion (i.e., ambiguous) (**Figure 1c–d**) face-voice pairings. The face-voice pairings remained congruent across identity. Each audio-visual stimulus was repeated three times such that a total of 240 audio-visual stimuli (120 congruent and 120 incongruent) were presented.

## Design and Procedure

### Experimental paradigm

It was requested that while trying to maintain central fixation during each trial, participants attend to the voice and the face of each actor. Participants were also asked to report and identify whenever the letter ‘B’ or ‘R’ appeared at the center of the screen following a select number of trials (48 in total, pseudo-randomly chosen) using 2 buttons on an ergonomic response box. This task-irrelevant response provided a way to monitor performance and was included to ensure that participants remained attentive to and centrally fixated upon each stimulus.

### Experimental trials

All trials began with a central black fixation cross sized 3×3 cm in diameter presented for 800 ms against a solid grey background. A centrally presented visual, binaural auditory, or audio-visual stimulus (binaural audio+central visual) next appeared on the screen for 800 ms. A solid grey screen then appeared for 1000 ms immediately following stimulus offset.

Each condition consisted of 120 trials, some of which could be further categorized by emotion: 120 unimodal voice trials (60 fear, 60 disgust); 120 unimodal face trials (60 fear, 60 disgust); and bimodal face-voice trials where the face was either congruent or incongruent with respect to the emotion expressed in the prosodic voice (240 bimodal trials; 60 AV congruent fear, 60 AV congruent disgust, 60 AV incongruent fear, 60 AV incongruent disgust). All stimuli were presented using Presentation software (Neurobehavioral Systems, San Francisco, CA), with trials randomized across participants to minimize habituation effects. The experiment was presented in 3 runs, with each run lasting approximately 8 minutes. Short breaks of a few seconds were provided to participants in between runs to minimize fatigue.

### Response trials

Forty-eight additional trials were included whereby a response was requested from the participant. During a response trial either a letter ‘B’ or ‘R’ appeared for 250 ms at the center of the screen directly following stimulus offset. Directly following letter offset a solid grey screen was presented for 750 ms. Response trials were always followed by a *dummy* trial (12 from each condition, pseudo-randomly chosen and counterbalanced across conditions). Due to potential motor response contamination, dummy trials were not used in the overall analysis of the data.

### Data acquisition

#### Magnetoencephalography

MEG data were acquired at the York Neuroimaging Centre. A 248-channel Magnes 3600 whole-scalp recording system (4-D Neuroimaging, San Diego, CA) with superconducting quantum interference device based first-order magnetometer sensors was used. For improved accuracy in co-registration with MRI data, each participant's head, nose, and eye orbit shapes were digitized prior to data acquisition using a stylus digitizer (Polhemus Isotrak). Three coils were placed at equally spaced locations across the forehead and two coils were positioned in front of the left and right ears of each participant to monitor head position prior to and following data acquisition. Participants were excluded from both sensor and source-level analyses when head movement values of 0.75 cm or greater were observed at three or more coils.

Participants were seated during the three runs of the experiment while their magnetic brain activity was continuously digitized. Data were sampled at a rate of 678.17 Hz (bandwidth 200 Hz) and filtered online using a direct current filter. Images were projected onto a screen located approximately 105 cm in front of participants and subtending a viewing angle of 8° for faces and 0.3° for letters. To minimize participant eye saccades, each face presented was small in size (5×10 cm). The letters displayed during response trials were sized 5 mm×1 cm to ensure that participants' maintained central fixation throughout each stimulus presentation. Auditory stimuli were presented via earphones (Etymotic Research ER30) at a comfortably audible level. Using a video camera located in the magnetic shielded room, participants were monitored throughout the experiment.

#### Magnetic resonance imaging

Standard structural MRI scans were obtained from each participant for co-registration with MEG data. Images were acquired on a 3-T scanner (HD Excite; General Electric) using a whole-head coil (8-channel high T-resolution brain array) and a 60-cm magnet. An automatic shim was applied before each scan to maximize magnetic-field homogeneity. One hundred and seventy-six sagittal slices (1-mm thick, 3-dimensional) parallel to midline structures covering the whole brain were imaged using an IR prepared Fast Spoiled Gradient Recalled (FSPGR) pulse sequence (repetition time = 6.6 ms, echo time = 2.8 ms, flip angle = 20°, and inversion time of 450 ms). The field of view was 290×290 mm, and the matrix size was 256×256, giving an in-plane spatial resolution of 1.13 mm.

After performing localizer and calibration scans, high-resolution T1 volumes were obtained with voxel dimensions of 1×1.13×1.13 mm. Three-dimensional gradient warping corrections and edge-enhancement filters were applied for enhanced distortion-elimination and improved co-registration of MRI and MEG data.

### Analysis of Imaging Data

#### Sensor-level analysis

Epochs were examined one-by-one to identify faulty sensors, and epochs containing swallow, eye saccade, blink, and electrical noise artefacts within the time window of interest (800 ms prior to stimulus onset and 800 ms after stimulus onset). Prior to performing analyses at the sensor and source-level, faulty sensors were zeroed. In addition, differences in overall DC level between sensors and artefact-contaminated epochs were removed. Two participants with unusable trials greater than 30% (∼165 or more epochs rejected) were excluded from further analyses.

To identify the frequency content underlying supra-additive activity, data were first analyzed in relation to the sensors surrounding a participant's head. Evoked activity in each condition was assessed for each participant and then for the group of participants. The brain's response to a presented stimulus can be locked in phase to the temporal onset of the stimulus and the analysis of evoked activity permits the identification of such phase-dependent information. Group-level differences are reported here.

The evoked signal was analyzed for the group of participants for all four conditions using a 1,000 ms time window (300 ms pre- and 700 ms post-stimulus onset). To examine the frequency content of the MEG signal, the data were averaged across participants for every condition, subjected to a fast Fourier transform (FFT), and then squared to ascertain the evoked power present in each condition for all 248 channels. Mains electricity components were removed and spectral leakage was minimized via application of a notch filter and Hanning window, respectively.

**Figure 2:**
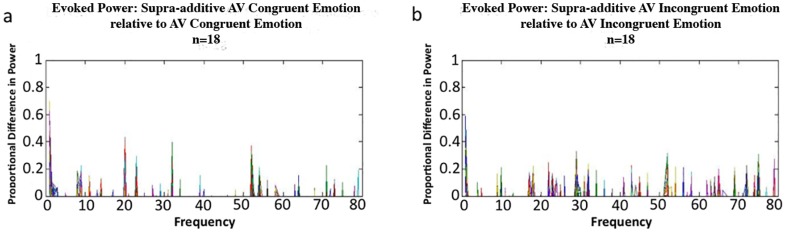
Group-level results showing proportional differences in supra-additive evoked power for (a) congruent and (b) incongruent emotion conditions (i.e., supra-additive AV divided by AV). Colored lines represent the group-level evoked response captured by every individual sensor that surrounds a participant's head.

We first calculated the means and standard deviations of the evoked power within each condition for the group of participants. Next, we summed the mean evoked power spectra of both unimodal conditions for all 248 sensor channels and then subtracted these values from the mean evoked power spectrum of the respective audio-visual condition to analyze the presence of supra-additivity at the group level in both congruent and incongruent audio-visual emotion conditions in accordance with Calvert et al. [13]. As observed in Hagan et al. [12], the theta (4–8 Hz) and alpha (8–13 Hz) frequency bands were observed to elicit the largest supra-additive responses. However, we were primarily interested in the relative contribution of each frequency bin to the overall supra-additive response. Therefore, we divided the supra-additive differences in power by the power spectra of the respective audio-visual condition (**Figure 2a–b**).

Frequencies lower than 3 Hz are potentially contaminated by external noise and therefore were not examined. Group-level supra-additive responses were not confined to any particular frequency band, but instead were observed from 3 Hz up to and including 80 Hz (**Figure 2a–b**). This observation guided our approach when performing the principal brain source-level analyses.

#### Source-level analysis

We co-registered the MEG data with the structural MRI scan of each participant, first by matching the surface of the head digitization maps to the surface of the structural MRI [29] and then by applying a spatial filter (i.e., *beamformer*) technique to localize those sources within the brain that related to the task performed [30]. The covariance matrix was constructed using a 1,300 ms time window (500 ms pre-stimulus plus 800 ms stimulus duration) and determined the spatial filter properties of the beamformer. Spatial filters estimate the amount that a single point within the brain grid volume contributes to a current source, independent from all other grid points [31]. For each participant, spatial filters were obtained for every point on a 5 mm grid covering the volume of the brain, in isolation of all other points. To provide maps of activity corresponding to each trial for every condition, the power at each grid point for specified frequency bands was calculated for every epoch and represented by a neuromagnetic activity index (NAI). Statistical non-parametric contrasts of these maps were created between conditions to localize sources of brain activity.

A beamformer was used to analyze evoked and induced broadband responses across space and time. Based upon our previous study [12] and the results observed at the sensor-level, neuromagnetic activity between 3 and 80 Hz, was selected for broadband analyses, with frequencies lower than 3 Hz excluded on the basis of insufficient numbers of waveform cycles in the 500 ms time windows of analyses.

Consistent with methods previously employed [12], [13], the NAIs of *active* and *passive* time windows were subtracted to produce an *active minus passive* contrast for all conditions. Passive periods were designated as neuromagnetic activity occurring for the 500 ms prior to each stimulus presentation whereas active periods were designated as neuromagnetic activity occurring during a 500 ms time window moving across the duration of each stimulus presentation. Active periods were therefore 0–500 ms, 50–550 ms, 100–600 ms, 150–650 ms, 200–700 ms, 250–750 ms, or 300–800 ms post-stimulus onset. To eliminate edge effects on passive and active time windows when filtering the data, a *buffer* of 600 ms was applied. As all stimuli were randomly presented, epochs from unimodal auditory (A) and unimodal visual (V) active minus passive contrasts were independently arranged according to their temporal order of presentation. The ordered epochs from the unimodal auditory contrasts were aligned and paired with the ordered epochs from the unimodal visual contrasts. Every epoch from the unimodal auditory condition was then summed with its unimodal visual epoch counterpart from the temporally ordered unimodal visual epochs (i.e., the first presented auditory stimulus was paired and summed with the first presented visual stimulus, and so on) to generate (A+V) epochs. This procedure was also used to estimate the variance across epochs. Supra-additive differences in power between the generated (A+V) epochs and the respective audio-visual epochs were assessed using non-parametric statistics.

Statistical non-parametric maps were generated from the supra-additive comparisons (i.e., AV>[A+V]) to localize the brain source of supra-additive increases and decreases in broadband power (3–80 Hz; see **Figure 2a–b**) and then with spectral filters for frequency bands commonly used in clinical studies (theta = 4–8 Hz; alpha = 8–13 Hz; beta = 13–30 Hz; gamma = 30–80 Hz). Statistical non-parametric maps were generated, first for the individual and then for the group in standard brain [Montreal Neurological Institute (MNI)] space.

Analyses at the level of the whole brain were performed but we restrict the discussion of our results to regions identified through previous studies of audio-visual speech and emotion integration [(i.e., superior temporal sulcus (STS) and adjacent regions]. Because of this selective region approach and because supra-additivity is already a stringent criterion for identifying crossmodal neural activity, we chose not to adjust the criterion for significance to account for family-wise error. Instead, as in our previous study [12] we used non-parametric t-tests to identify significant (p<.05 whole brain level, uncorrected) supra-additive increases and decreases in power for incongruent emotion, congruent emotion, congruent disgust and congruent fear conditions within overlapping 500 ms time-windows across the duration of the stimulus (i.e., 0–500 ms, 50–550 ms, 100–600 ms, 150–650 ms, 200–700 ms, 250–750 ms, 300–800 ms).

#### Masking procedure

Consistent with Calvert et al. [13] activations in supra-additive areas were also required to be at least minimally active during both unimodal conditions. Both increases and decreases in power can be observed using beamforming approaches and both are suggested as important neural phenomena when trying to understand how the brain functions [32]. The criterion of minimal activity in both unimodal auditory and unimodal visual conditions can therefore be satisfied according to four alternative response patterns. That is, voxels displaying supra-additivity could produce either: (1) increases in power (i.e., t-values greater than 0) in both auditory and visual conditions; (2) decreases in power (i.e., t-values less than 0) in both auditory and visual conditions; (3) an increase in power in the unimodal auditory condition and a decrease in power in the unimodal visual condition; and lastly, (4) an increase in power in the unimodal visual condition and a decrease in power in the unimodal auditory condition.

The response in the primary visual cortex was observed as a decrease in power for the V condition, whereas the response in the primary auditory cortex was observed as an increase in power for the A condition when independently compared with the fixation cross baseline. Notably, this response pattern is the same as that observed during our previous research [12] and mirrors findings in non-human primates [10]. As the main aim was to minimize supra-additive responses in unisensory areas, mask alternative 3 (detailed above) was adopted and beamformed to localize those voxels satisfying the two criteria for auditory and visual conditions. In the instance where both criteria were not satisfied in a particular voxel, the contribution of that voxel to the overall calculation of statistical power was not considered.

## Results

Following the procedures developed by Hagan et al. [12], a two-stage approach was taken when analyzing the MEG data. First sensor-level data were analyzed to examine the frequency content of the supra-additive signal, and second, source-level data were used to localize the neural sources underlying the supra-additive signal(s). More detailed description of the sensor and source-level analysis procedures are provided in Hagan et al. [12]. Results from the separate analysis of fear and disgust trials are presented in Information S1.

The results detailed below focus on the group-level supra-additive broadband response observed in STS and nearby brain regions within 500 ms time bins at 50 ms intervals across the duration of the presented stimulus. Findings representing supra-additive increases in power (AV>[A+V]) are described, as these results are in line with the *a priori* examination of supra-additivity. However, a thorough analysis of the data also revealed supra-additive decreases in broadband power (AV<[A+V]). For the sake of transparency with regard to all results observed, we present these supra-additive decreases in broadband power, whenever they occurred, in the tables below. Supra-additive increases and decreases in power within different frequency bands are available in Information S1.

### AV Congruent Emotion>(Auditory Emotion+Visual Emotion)

#### Broadband (3–80 Hz)

**Figure 3:**
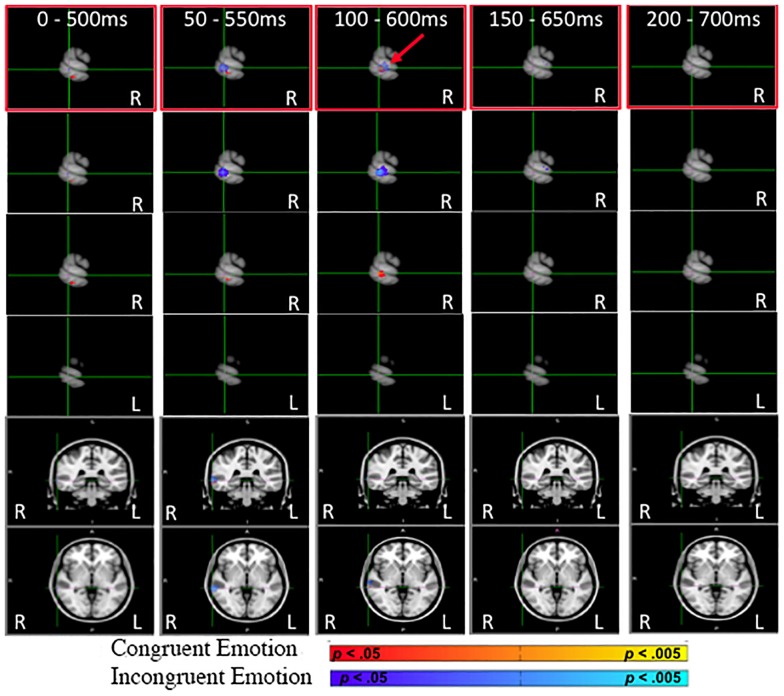
Group data showing supra-additive increases in power observed broadband for the AV Congruent Emotion and AV Incongruent Emotion conditions across the first 700 ms of the presented stimulus. Each column depicts the supra-additive response observed in a 500 ms time window beginning at 0 ms, with time windows of subsequent columns increasing in increments of 50 ms up to 200 ms. The top row depicts the sagittal plane depicting overlapping supra-additive activation from both the incongruent emotion and congruent emotion conditions in right STS. Note that this overlap is especially pronounced in the 100–600 ms time window (indicated by the red arrow). The second row depicts the sagittal plane showing supra-additive activation observed in the incongruent emotion condition only whereas the third row shows the sagittal plane indicating the supra-additive activation observed in the congruent emotion condition only. The fourth row depicts the sagittal plane showing a lack of supra-additive activation observed in left STS for either condition. The fifth and sixth rows depict activation across axial and coronal planes, respectively. Crosshairs are placed at MNI coordinate in MTG (±68, −36, −2) with activation thresholded from *p*<.05 – *p*<.005. T-values observed at 68, −36, −2 are as follows for congruent/incongruent emotion conditions: 0–500 ms = 0.88/1.72; 50–550 ms = 1.58/2.35; 100–600 ms = 2.23/3.14; 150–650 ms = 0/0; 200–700 ms = 0/0.

Clusters representing group-level significant supra-additive increases in power were observed broadband for the congruent emotion condition. Consistent with the activity observed in Hagan et al. [12], one cluster peaked in right middle temporal gyrus (MTG [BA 21]), with activity spreading to encompass right STS and right superior temporal gyrus (STG) by 150 ms (**Figure 3** **;** **Table 1**). Note that significant supra-additivity in right STS, right STG, and right MTG occurred in all time windows until the 150–650 ms time window (**Table 1**), and was absent in all subsequent time windows (i.e., 200–700 ms onwards). Also note that while significant supra-additive broadband activity occurred in anterior regions of left STG, this activity was constrained to the 0–500 ms time window (**Table 1**). Other clusters of significant supra-additive increases in power are presented in **Table 1**.

**Table 1:** Coordinates in MNI space and associated peak t-scores showing the significant differences (one-tailed) in power for the main effect of congruent audio-visual emotion minus (auditory emotion+visual emotion).

*Brain Regions*				Coordinates		
	BA	P Value	T Score	X	Y	Z
***Broadband (3–80 Hz)***						
***AV Congruent– (A+V)***						
**0–500 ms**						
R Middle Temporal gyrus, R Inferior Temporal gyrus	21	<.020	2.23	64	−30	−16
R Temporal pole	21	<.045	1.82	−56	10	−26
L Anterior Superior Temporal gyrus	38	<.050	1.79	−46	4	−22
***Broadband (3–80 Hz)***						
***AV Congruent – (A+V)***						
**50–550 ms**						
L Temporal pole	−	<.020	2.22	−40	10	−22
R Middle Temporal gyrus	21	<.025	2.16	64	−30	−12
L Temporal pole	38	<.025	2.13	−50	14	−26
R Temporal pole	38	<.035	1.93	60	14	−12
R Temporal pole	21	<.040	1.89	54	10	−36
***Broadband (3–80 Hz)***						
***AV Congruent– (A+V)***						
**100–600 ms**						
R Middle Temporal gyrus, R Superior Temporal sulcus, R Superior Temporal gyrus	21	<.020	2.31	70	−30	−12
R Middle Temporal gyrus	21	<.020	2.29	64	−36	−2
L Insula	38	<.030	2.10	−40	10	−16
L Medial Prefrontal cortex	10	<.030	2.07	−6	64	18
R Temporal Pole	21	<.035	1.94	54	10	−36
***Broadband (3–80 Hz)***						
***AV Congruent – (A+V)***						
**150–650 ms**						
L Temporal Pole	38	<.020	2.23	−50	20	−22
R Superior Temporal gyrus	42	<.050	1.77	64	−20	8
***Broadband (3–80 Hz)***						
***AV Congruent – (A+V)***						
**200–700 ms**						
R Precentral gyrus	6	<.050	1.74	64	0	28
***Broadband (3–80 Hz)***						
***AV Congruent– (A+V)***						
**250–750 ms**						
R Precentral gyrus	6	<.045	1.79	64	0	28
***Broadband (3–80 Hz)***						
***AV Congruent– (A+V)***						
**300–800 ms**						
n.s.						
**BA = Brodmann Area; L = Left; R = Right; n.s. = No significant areas**						

Positive t-scores reflect significant increases in power whereas negative t-values reflect significant decreases in power.

### Frequency Band Analysis

#### Theta (4–8 Hz)

Only three clusters were observed in the theta frequency band representing significant supra-additive increases in power for the AV Congruent Emotion condition. However, none of these clusters were located in STG or surrounding areas. Regions displaying significant supra-additive increases and decreases in power observed in the theta frequency band are reported in Table S5 in Information S1.

#### Alpha (8–13 Hz)

Several clusters representing significant supra-additive increases in power for the AV Congruent Emotion condition were observed in the alpha frequency band. Several clusters peaked in right STG and surrounding regions (BA 22, BA 38) with activation spreading to encompass right STS. Regions displaying significant supra-additive increases and decreases in power observed in the alpha frequency band for the AV Congruent Emotion condition are reported in Table S6 in Information S1.

#### Beta (13–30 Hz)

No clusters representing supra-additive increases in power for the AV Congruent Emotion condition were observed during any time window for the beta frequency band.

#### Gamma (30–80 Hz)

Several clusters representing supra-additive increases in gamma power were observed for the AV Congruent Emotion condition. One cluster was observed peaking in right STG (BA 22) with activation spreading to encompass right planum temporale and right postcentral gyrus (Table S7 in Information S1).

### AV Incongruent Emotion>(Auditory Emotion+Visual Emotion)

#### Broadband (3–80 Hz)

Across the group of participants, significant supra-additive increases in broadband power were observed for the incongruent emotion condition. Notably one supra-additive cluster was observed in precisely the same area displaying supra-additive increases in power during the congruent emotion condition, with overlap particularly prominent in the 100–600 ms time window. This cluster peaked in right MTG (BA 21) with activation extending to include STS and STG across a similar timeframe to that observed during the congruent emotion condition (**Figure 3** **;** **Table 2**). No significant supra-additive increases in broadband power were observed in temporal lobe areas in the left hemisphere. **Table 2** presents the supra-additive increases and decreases in power observed for the incongruent emotion condition across the group of participants. As no statistically significant supra-additivity was observed in late time windows for either the AV incongruent or AV congruent emotion conditions, **Figure 3** only depicts the activity observed through the first 700 ms as opposed to the entire 800 ms of stimulus presentation.

**Table 2:** Coordinates in MNI space and associated peak t-scores showing the significant differences (one-tailed) in power for the main effect of incongruent audio-visual emotion minus (auditory emotion+visual emotion).

*Brain Regions*				Coordinates		
	BA	P Value	T Score	X	Y	Z
***Broadband (3–80 Hz)***						
***AV Congruent– (A+V)***						
**0–500 ms**						
R Temporal pole	21	<.030	2.03	60	10	−32
R Middle Temporal gyrus	21	<.050	1.76	70	−36	−2
***Broadband (3–80 Hz)***						
***AV Congruent – (A+V)***						
**50–550 ms**						
R Middle Temporal gyrus, R Superior Temporal sulcus, R Superior Temporal gyrus	21	<.020	2.35	64	−36	−2
***Broadband (3–80 Hz)***						
***AV Congruent– (A+V)***						
**100–600 ms**						
R Middle Temporal gyrus, R Superior Temporal sulcus, R Superior Temporal gyrus	21	<.005	3.15	70	−36	−2
R Temporal pole		<.035	1.97	54	10	−36
***Broadband (3–80 Hz)***						
***AV Congruent – (A+V)***						
**150–650 ms**						
R mid-anterior Superior Temporal gyrus	21	<.020	2.25	70	−20	4
***Broadband (3–80 Hz)***						
***AV Congruent – (A+V)***						
**200–700 ms**						
R mid-anterior Superior Temporal gyrus	42	<.030	2.03	70	−16	8
***Broadband (3–80 Hz)***						
***AV Congruent– (A+V)***						
**250–750 ms**						
n.s.						
***Broadband (3–80 Hz)***						
***AV Congruent– (A+V)***						
**300–800 ms**						
L Lateral Frontal Pole	47	<.040	−1.88	−56	40	−2
**BA = Brodmann Area; L = Left; R = Right; n.s. = No significant areas**						

Positive t-scores reflect significant increases in power whereas negative t-values reflect significant decreases in power.

### Frequency Band Analysis

#### Theta (4–8 Hz)

One large cluster representing significant supra-additive increases in power in the theta frequency band for the AV Incongruent Emotion condition was observed peaking in right MTG (Table S8 in Information S1). This cluster first appeared in the 150–650 ms time window, spreading to encompass STS and STG over time, and remaining through to stimulus offset. Regions displaying significant supra-additive increases and decreases in power observed in the theta frequency band are reported in Table S8 in Information S1.

#### Alpha (8–13 Hz)

Several clusters representing significant supra-additive increases in power were observed in the alpha frequency band. One cluster peaked in right posterior STG (BA 22) with activation spreading anteriorly to encompass more mid sections of STG (BA 42) over time. Other regions displaying significant supra-additive increases and decreases in power observed in the alpha frequency band are reported in Table S9 in Information S1.

#### Beta (13–30 Hz)

No clusters representing supra-additive increases in power for the AV Incongruent Emotion condition were observed during any time window for the beta frequency band.

#### Gamma (30–80 Hz)

A few clusters representing supra-additive increases in gamma power were observed for the AV Incongruent Emotion condition. One cluster was observed peaking in right STG (BA 21) during the 200–700 ms time window. Another cluster was observed peaking in left MTG during the 250–750 ms time window, with activation spreading to encompass left STS and left STG (Table S10 in Information S1).

## Discussion

We previously paired static pictures with non-verbal vocal expressions and observed supra-additive responses in right posterior STS [12]. The current study extends our previous study of crossmodal emotional integration by examining supra-additive responses in STS for dynamic depictions of facial and vocal prosodic speech stimuli. Our main question was whether these ecologically valid dynamic emotional displays would elicit supra-additive responses in right STS or nearby regions akin to findings from previous studies of crossmodal emotional processing and affective prosody [12], [14], [25], or whether the use of speech stimuli would engage left STS more strongly [7], [13]. When combining the results observed at sensor- and source-levels, we demonstrate evoked supra-additive broadband activation in right STS for both congruent and incongruent emotional displays. We interpret these findings in the context of facial emotion perception and the broader role of STS in crossmodal signal integration.

Supra-additive broadband activity for congruent and incongruent emotional displays was observed to overlap in right STS. In line with findings of a rapid-onset for crossmodal emotion integration [1], [12], [27], supra-additive broadband activity was observed to occur in right STS within 250 ms for incongruent and 200 ms for congruent affective speech displays. While our incongruent emotion condition did elicit a temporal delay of 50 ms in the audio-visual response, this minimal delay is not inconsistent with other crossmodal studies reporting similar findings [33]. Van Wassenhove et al [34] showed that the auditory ERP (N1/P2) occurred earlier only when accompanied by congruent orofacial movements. Although EEG is not optimal for detecting the neural source of signals, STS was suggested as a possible locus for the temporal facilitation effect observed. Arnal et al. [35] later localized the source of the M100 (MEG equivalent of N1/P2) temporal facilitation to middle temporal visual areas (MT/V5) yet also suggested that STS was part of an indirect pathway involved in congruency-related phase resetting. We attribute the minimal difference in time course between congruent and incongruent conditions to the minimal temporal incongruence across the auditory and visual video streams of each incongruent emotion stimulus item with the incongruency effect more akin to an emotional McGurk effect. While studies examining behavioral evidence of fusion across sensory streams report a 200 ms offset window for the temporal integration of AV speech [36], neuroimaging studies show that even a temporal misalignment of 400 ms is insufficient to produce significant reductions in right STS responses [19]. This may be because, like incongruent human speech cues, incongruent human emotional speech cues arise through conflicting phonetic information rather than conflicting spatio-temporal information, and this conflict in phonetic information appears to be somewhat resistant to adaptation and alteration [37]. Interestingly, Miller and D'Esposito [38] identified STS as a region more involved in the phonetic/sensory correspondence relative to the spatio-temporal correspondence of speech processing. Findings of supra-additivity in STS within 250 ms of crossmodal stimulus presentation are in line with our previous MEG study of nonverbal emotional displays [12] and suggest that emotion integration occurs quickly irrespective of congruence in multisensory display. Furthermore, electrophysiological studies of crossmodal integration demonstrate audio-visual interactions before 200 ms [1], [26] and ERP research shows enhancements to the auditory N1 component as early as 110 ms after crossmodal emotion presentation [27].

Interestingly, our observation of supra-additive responses in right STS for incongruent emotional speech is different from other studies showing sub-additive activation in right STS for mismatches in the verbal content (and by default, in the temporal correspondence) of audio-visual speech [13]. The verbal content of the auditory and visual streams of the stimuli used in the present paper were also congruent (i.e., the spoken word “hat” was paired with a video display of DM or GL speaking the word “hat”), however, the acoustical elements of the auditory and visual emotive streams were incongruent resulting in emotional ambiguity. While previous studies have not assessed whether incongruent stimuli elicit supra-additive signals in STS, some studies have noted greater activity for incongruent relative to congruent AV speech stimuli in STS [20] and other cortical regions [19]. Of note is that Jones and Callan [19] showed significant activation in bilateral STG for incongruent AV speech stimuli (i.e., McGurk) irrespective of the temporal offsetting between A and V modalities. The principle of inverse effectiveness during crossmodal integration holds that the crossmodal response can amplify information from degraded signals relative to non-degraded signals [39], and this principle has been tested by adding dynamic noise to A and V conditions to elicit supra-additive responses in STS [40]. In the current study, however, neither sensory channel was degraded. Either of the unimodal stimuli might have provided interpretable information, but their combination either created perceptual incongruence or allowed for faster resolution of congruent information, consistent with the *analysis-by-synthesis* model that perceptual outcomes are dependent on the redundancy of sensory information across perceptual streams proposed by van Wassenhove et al. [34]. That supra-additivity was found under these circumstances is in line with it being a mandatory feature of STS responses.

Emotion perception and recognition are crucial social skills important for successful negotiations with peers. It is therefore possible that the utility of emotional inference takes precedence over precision in emotion identification [41]. This interpretation of emotion perception would suggest that facial and vocal integration is a mandatory process, a view put forward by De Gelder and Vroomen [42]. Our data showing that facial-vocal conditions elicit supra-additive response enhancements irrespective of congruence appear to support such a view.

Brain regions other than STS may also be important sites for dynamic crossmodal emotion integration. Although we had no a priori hypothesis with regard to the temporal pole, supra-additive response enhancements were observed in this region for both congruent and incongruent emotional speech conditions. This is in contrast to other brain regions displaying broadband supra-additive increases in power that were sensitive to the emotional congruence across speech streams. These regions included the insula, medial prefrontal cortex and precentral gyrus for the congruent emotional speech condition and the lateral frontal pole for the incongruent emotional speech condition. Further research examining the precise role of these regions in the integration of audio-visual emotive speech is warranted.

One potential criticism of the current study is that the visual nature of the behavioral task utilized only ensured that participants pay attention to the visual stream of each stimulus. We chose this behavioural task because it was identical to that used in our previous study [12]. Nonetheless, studies of crossmodal object recognition demonstrate that multisensory effects are sometimes critically dependent on attention to both (as opposed to one) sensory modalities [43]. Of note is that Talsma et al., [43] observed no supra-additive responses when participants attended to a single modality, which was interpreted as suggesting that attention to both A and V modalities can be necessary for the observation of facilitation effects associated with AV integration. While our data showed supra-additive responses in right STS for both incongruent and congruent AV emotion conditions, it is possible that we would have observed a different pattern of facilitation effects had we incorporated both auditory and visual behavioral tasks. Future studies adopting this approach are therefore warranted. For the time being, though, a parsimonious interpretation of our findings is that resolution of an audio-visual emotional signal, whether incongruent or congruent, requires engagement of right STS, and is reflected in supra-additive activity. Our data are in favor of the early, and possibly automatic, integration of acoustic and visual signals of emotion irrespective of congruence or ambiguity in perceptual content.

### Supra-additivity within frequency bands

Akin to studies of crossmodal integration conducted in non-human primates [10] and humans [12], broadband responses in STS occurred in regions exhibiting decreases in power to visual stimuli and increases in power to auditory stimuli when compared independently with the fixation cross baseline. When spatial filters were applied to examine the frequency content of the supra-additive signal, small yet distinct clusters of supra-additive increases in gamma, alpha, and theta power were observed in right STG, STS and surrounding regions for both incongruent and congruent emotional AV displays. Interestingly, theta activity (4–8 Hz) appeared to overlap the most with the supra-additive broadband response observed in right STS, which is different to the observation of overlapping gamma and broadband activity reported in our previous study [12]. Notably, significant supra-additive increases in theta power were observed in right STS and nearby temporal regions (see Information S1) only for the incongruent AV emotion condition. The use of vocal prosody as opposed to nonverbal vocal expressions may, in part, help to explain this difference as the most prominent envelope frequencies of human speech typically fall within the theta range [44] and oscillations in the theta band are commonly observed in auditory cortex [45]. Unsurprisingly, the theta band has been suggested to coordinate the auditory modality of AV speech [46]. Luo and Poeppel [47] suggest that changing speech patterns might be tracked by oscillatory theta activity. Indeed, an exploratory MEG study of audio-visual speech observed greater activity in the theta frequency band, as compared to alpha and gamma frequency bands, during auditory stimulus presentation [46]. Fingelkurts and colleagues [46] therefore suggested that the theta frequency band could reflect coordinated processing related to specific elements within the auditory stream of a crossmodal stimulus. Notably, the emotional voice stimuli presented in the incongruent emotion and congruent emotion conditions were entirely the same, albeit differentially paired to the emotional face stimuli. Thus, while an auditory processing account of theta activity coincides with the observation of supra-additive increases in power for the incongruent emotion condition, that account does not entirely reconcile with the observation of no significant supra-additive increases in theta power in STS during the congruent emotion condition.

Significant supra-additive increases in beta activity were neither observed for the incongruent emotion nor the congruent emotion AV conditions. Previous studies implicate the beta frequency band in processing sensory-motor information [48]. The absence of supra-additive increases in beta activity may reflect that participants were not required to perform a task related to the crossmodal presentation of stimuli.

Supra-additive increases in power in the alpha frequency band were observed in STS and nearby regions for both congruent emotion and incongruent emotion AV conditions. The alpha frequency band has been suggested to coordinate the visual modality of an AV stimulus [46]. Alpha activity has also been suggested to reflect the attempt to suppress or inhibit information arising from particular neural regions [49]. As both explanations of alpha activity are plausible within the present dataset, future studies are needed before determining whether the supra-additive increases in alpha activity we observe is reflective of the visual portion of an audio-visual stimulus [46] or the functional inhibition of cortical regions [49].

The gamma frequency band was also observed to elicit small but significant increases in power in right STG and surrounding regions during the congruent and incongruent AV emotion conditions. The *representational hypothesis* of gamma asserts that gamma activity serves as a signal that links both proximal and distal cortical regions during object representation [50]. A review by Senkowski and colleagues [51] highlights this aspect of gamma, suggesting that gamma may serve to bind congruent multisensory signals across the cortex. These suggestions are in line with the hypothesis put forward by Singer and Gray [52] that the gamma band may serve to bind the features that comprise a visual stimulus and could also underlie other forms of sensory integration. As supra-additive increases in gamma power were observed at earlier latencies for the congruent emotion relative to the incongruent emotion condition, it is possible that congruency across sensory modalities enhances or modulates supra-additive responses in STG and surrounding temporal regions, although the present study alone does not permit one to draw definitive conclusions about the role of supra-additive increases in gamma band activity.

Research investigations into the precise role(s) of specific frequency bands are ongoing, and so it is not possible to ascribe with certainty any particular role(s) to alpha, theta, beta or gamma frequency bands. Further research targeted at independently monitoring each frequency band during crossmodal emotion presentation would be necessary to elucidate the mechanisms attributable to the neural oscillatory signals underlying crossmodal perception and integration. Of general interest and utility to researchers using MEG is the reliability with which one can ascribe a particular frequency band(s) to a specific brain region or set of brain regions implicated in a neural system. Consistent divisions of frequency space may therefore be necessary before researchers can reliably compare results obtained in different frequency bands across studies.

### Study Limitations

Two potential limitations of the current experiment are that the visual and acoustic elements of the AV stimuli were synchronized, which may have minimized the potential for detecting supra-additive response enhancements [53] and that the study did not include comparison conditions of neutral facial expression and neutral vocal intonation. These conditions were considered for the experiment, but left out of the final design. Our previous study [12] had shown no evidence of right STS involvement in the audio-visual integration of minimally congruent neutral stimuli. We therefore considered it important to investigate effects of audio-visual emotion congruence in the present experiment, and because MEG experiments necessarily involve substantial numbers of trials per condition, this left us with no scope for including additional neutral conditions. Adding neutral condition trials would also have substantially lengthened the experiment, and we were concerned to minimize participant fatigue. For this reason we opted to have a substantial number of trials in each condition while including only those conditions essential for investigating audio-visual emotion integration.

### Study Conclusions

In summary, incongruent and congruent displays of dynamic facial and vocal emotive speech signals elicit evoked supra-additive broadband power at the sensor-level. When the neural sources of this supra-additivity were analyzed, both congruent and incongruent emotion conditions elicited a significant supra-additive increase in broadband power in right STS within the first 250 ms of crossmodal stimulus presentation. Notably, supra-additive broadband activity was observed in overlapping temporal regions despite that the stimuli presented in the congruent emotion and incongruent emotion conditions were differentially paired. This supra-additive broadband response to verbal AV emotion showed a comparable location and time course to that found for nonverbal AV emotional displays [12], underscoring the importance of the right STS region to crossmodal signal integration for the interpretation of emotion.

## Supporting Information

Information S1 Tables S1, S2, S3, S4, S5, S6, S7, S8, S9, S10
(DOC)
