# Supplementary material for: Involvement of Right STS in Audio-Visual Integration for Affective Speech Demonstrated Using MEG
Source: PLoS One. 2013 Aug 12;8(8):e70648. doi: 10.1371/journal.pone.0070648 (PMC3741276; doi:10.1371/journal.pone.0070648)
Supplement: Information S1 — Tables S1, S2, S3, S4, S5, S6, S7, S8, S9, S10 (DOC) [file pone.0070648.s001.doc]

**Supporting Information**

**Supporting Materials and Methods**

Video Stimuli Creation

| **Word** | **ANEW Word Number** | **Mean Valence** | **SD Valence** | **Mean Arousal** | **SD Arousal** | **Mean Dominance** | **SD Dominance** | **Word Frequency** | **Word Letter Length** |
| --- | --- | --- | --- | --- | --- | --- | --- | --- | --- |
| bench | 655 | 4.61 |  | 3.59 |  | 4.68 |  | 35.00 | 5.00 |
| **bowl** | **49** | **5.33** |  | **3.47** |  | **4.69** |  | **23.00** | **4.00** |
| bus | 541 | 4.51 |  | 3.55 |  | 4.84 |  | 34.00 | 3.00 |
| **chair** | **66** | **5.08** |  | **3.15** |  | **4.56** |  | **66.00** | **5.00** |
| clock | 688 | 5.14 |  | 4.02 |  | 4.67 |  | 20.00 | 5.00 |
| **cord** | **698** | **5.10** |  | **3.54** |  | **5.00** |  | **6.00** | **4.00** |
| cork | 699 | 5.22 |  | 3.80 |  | 4.98 |  | 9.00 | 4.00 |
| egg | 736 | 5.29 |  | 3.76 |  | 4.49 |  | 12.00 | 3.00 |
| fork | 560 | 5.29 |  | 3.96 |  | 5.74 |  | 14.00 | 4.00 |
| **fur** | **180** | **4.51** |  | **4.18** |  | **4.32** |  | **13.00** | **3.00** |
| hat | 783 | 5.46 |  | 4.10 |  | 5.39 |  | 56.00 | 3.00 |
| **hay** | **784** | **5.24** |  | **3.95** |  | **5.37** |  | **19.00** | **3.00** |
| ink | 229 | 5.05 |  | 3.84 |  | 4.61 |  | 7.00 | 3.00 |
| **jug** | **829** | **5.24** |  | **3.88** |  | **5.05** |  | **6.00** | **3.00** |
| **lawn** | **841** | **5.24** |  | **4.00** |  | **5.37** |  | **15.00** | **4.00** |
| seat | 380 | 4.95 |  | 2.95 |  | 4.84 |  | 54.00 | 4.00 |
| **spray** | **992** | **5.45** |  | **4.14** |  | **5.12** |  | **16.00** | **5.00** |
| stove | 1001 | 4.98 |  | 4.51 |  | 5.36 |  | 15.00 | 5.00 |
| **truck** | **577** | **5.47** |  | **4.84** |  | **5.33** |  | **57.00** | **5.00** |
| **vest** | **1026** | **5.25** |  | **3.95** |  | **5.09** |  | **4.00** | **4.00** |
|  |  |  |  |  |  |  |  |  |  |
|  | **MEAN** | **5.12** | **0.29** | **3.86** | **0.43** | **4.98** | **0.37** | **24.05** | **3.95** |

Audio-visual stimuli were recorded in an anechoic room with a digital video camera. Two speakers (DM, GL) were chosen out of a set of seven original speakers (all non-professional actors) with regional British English accents. The head and shoulders were captured in each video frame. Each actor spoke 20 monosyllabic words with fearful or disgusted expression and affective intonation. The 20 words were selected from the Affective Norms for English Words (ANEW; [1]**; Table** S1). Words with low scores in both valence and arousal (i.e., affectively neutral) were selected to permit greater focus on participant responses to the way in which the words were spoken (both visually and acoustically) as opposed to the semantic content of the words. The selection of neutral words also limited the possibility that an additional level of incongruence would be perceived across channels such that signals arising through the emotional content conflicted little with the semantic content of the spoken word.

**Table S1.** Words selected from ANEW list and corresponding mean valence, arousal and dominance ratings in addition to word frequency (taken from Kucera and Francis [2] norms) and letter lengths. Standard deviations (SD) for the valence, arousal and dominance ratings are also included. The words selected for use in the final video stimulus set are indicated in bold.

The video of each of the words spoken by each actor was individually cut, edited, selected for best depiction of audio-visual expression, and subsequently rendered to make independent video clips of the best audio-visual expression of each word 800ms in duration (20 frames) using Adobe Premiere. Where video editing was required, the editing was performed on a frame-by-frame basis and mainly consisted of actor centering or frame speeding and slowing to arrive at an 800ms clip capturing the spoken word in its entirety. The experimenter and a naïve colleague of the experimenter selected the best depiction of audio-visual stimuli with the criteria that the clips should be free from blink and eye movements, and should best characterize the intended emotion in both facial and vocal modalities. Occasionally, the auditory track and visual track of two separate clips from the same actor were amalgamated to generate a new audio-visual clip to ensure for the best visual and auditory emotional depictions of a spoken word. In this instance, the new audio-visual clip was edited on a frame-by-frame basis to ensure correct visual-temporal alignment of the auditory and visual portions of the stimulus. If correct visual-temporal alignment of the auditory and visual portions of the stimuli could not be obtained (i.e., when a single 40ms frame was offset), then the better of the original two audio-visual stimuli was instead used.

Files were saved individually as a multiplexed .mpeg file. The visual portions of each clip were also saved as an .mpeg file without the auditory accompaniment thus creating the unimodal visual stimuli (40 fear, 40 disgust, 40 neutral stimuli; 25 frames per second [fps] sampling rate). The auditory track of each video clip was sampled at 44kHz and saved as an uncompressed 16-bit .mpeg file with the visual track being a solid grey screen background (the same background as that used in the audio-visual stimuli). Auditory stimuli (120 in total across the 2 actors) were also saved using a sampling rate of 25 fps.

Video Stimuli Validation

Each stimulus was rated for intensity of fear and disgust over the course of two consecutive days by nine raters (4 male; mean age ± standard deviation [SD] = 24.20 ± 5.19; range = 20.07 – 37.33 years) who were right-handed, with normal hearing and vision, and without a history of psychiatric illness and neuropsychological injury. Written informed consent was obtained from all participants. Ethical approval was granted by the Department of Psychology at the University of York.

*Ratings Procedure*

All stimuli were presented using E-Prime. Raters were first presented with a stimulus and then asked to rate the intensity of fear (or disgust) in the stimulus for each of the stimuli using a 7-point Likert scale (1 = not at all fearful/disgusted and 7 = very fearful/disgusted). The order in which the two emotions were rated was counterbalanced across raters. The ratings were performed at the pace of the rater. Choice of either a monetary stipend or course credit was given to each rater upon completion of the rating procedure. Rating responses and RT were obtained for each person performing the ratings.

*Video Stimuli Selection*

Mean rating responses were used to select the best-rated and distinctive video depictions of fear and disgust emotional expressions. Two independent criteria were applied simultaneously. These criteria were that every video should be 1) highly recognizable as the intended emotion (i.e., ratings of 4 or higher on the Likert scale for audio-visual and corresponding unimodal auditory & unimodal visual stimuli for each word) and, 2) highly distinguishable from the unintended emotion (i.e., disgust ratings discrepant from fear ratings by a difference of 2 or greater). These criteria identified ten ANEW words (**Table S1**) spoken by three male actors (DM, MH & GL) as stimuli suitable for use in subsequent experiments (mean arousal ± SD = 3.91 ± 0.46; mean valence ± SD = 5.19 ± 0.27). Stimuli from actors DM and GL were rated as being the most distinct from each other in both visual and auditory domains by four independent raters. Therefore DM and GL were the actors selected for use in the current neuroimaging experiment.

**Table S2** shows the mean disgust intensity ratings given by participants for the fear and disgust stimuli selected.

**Table S2.** Mean disgust intensity rating responses and response times for stimuli selected from actors DM and GL.

|  |  |  | **Mean Disgust Rating** | | | **Mean Rating RT** | | |
| --- | --- | --- | --- | --- | --- | --- | --- | --- |
|  |  |  | **(1=not at all disgusted, 7=very disgusted)** | | | **(in ms)** | | |
| **Stimulus Type** | **Actor** | **Word** | **Audio-visual** | **Auditory** | **Visual** | **Audio-visual** | **Auditory** | **Visual** |
| disgust | DM | bowl | 6.56 | 5.11 | 6.67 | 553.89 | 736.67 | 327.44 |
| disgust | GL | bowl | 6.44 | 5.78 | 5.89 | 507.44 | 822.33 | 523.22 |
| disgust | DM | chair | 6.44 | 4.56 | 6.00 | 331.89 | 1037.33 | 543.44 |
| disgust | GL | chair | 6.56 | 5.78 | 6.78 | 389.33 | 1121.22 | 376.22 |
| disgust | DM | cord | 6.78 | 4.44 | 6.67 | 350.11 | 807.89 | 2267.11 |
| disgust | GL | cord | 6.22 | 5.11 | 5.67 | 223.78 | 594.44 | 652.44 |
| disgust | DM | fur | 6.78 | 5.67 | 6.56 | 452.44 | 845.44 | 369.67 |
| disgust | GL | fur | 6.67 | 5.11 | 6.33 | 350.44 | 945.67 | 627.89 |
| disgust | DM | hay | 6.67 | 4.00 | 6.44 | 372.56 | 1059.00 | 394.89 |
| disgust | GL | hay | 6.67 | 4.33 | 6.67 | 428.89 | 881.89 | 450.78 |
| disgust | DM | jug | 6.67 | 4.67 | 6.56 | 378.22 | 540.33 | 453.78 |
| disgust | GL | jug | 6.00 | 4.11 | 5.89 | 493.00 | 865.33 | 425.89 |
| disgust | DM | lawn | 6.33 | 4.22 | 6.22 | 544.78 | 992.00 | 477.67 |
| disgust | GL | lawn | 6.44 | 4.44 | 6.44 | 525.78 | 1110.44 | 342.11 |
| disgust | DM | spray | 6.56 | 5.56 | 6.78 | 428.67 | 709.11 | 349.22 |
| disgust | GL | spray | 6.22 | 5.56 | 6.33 | 512.22 | 466.11 | 396.44 |
| disgust | DM | truck | 6.00 | 6.00 | 6.33 | 380.00 | 750.33 | 386.44 |
| disgust | GL | truck | 6.56 | 5.22 | 6.56 | 413.11 | 727.56 | 421.11 |
| disgust | DM | vest | 6.33 | 4.44 | 6.56 | 302.89 | 407.00 | 389.33 |
| disgust | GL | vest | 6.00 | 4.22 | 6.22 | 357.67 | 576.44 | 581.78 |
|  |  | **MEAN** | **6.45** | **4.92** | **6.38** | **414.86** | **799.83** | **537.84** |
|  |  |  |  |  |  |  |  |  |
| fear | DM | bowl | 1.89 | 1.67 | 1.78 | 406.67 | 513.11 | 711.33 |
| fear | GL | bowl | 1.89 | 2.00 | 2.00 | 344.00 | 710.44 | 575.67 |
| fear | DM | chair | 1.78 | 1.67 | 2.00 | 444.11 | 429.11 | 366.89 |
| fear | GL | chair | 1.78 | 1.67 | 1.78 | 529.44 | 682.44 | 631.11 |
| fear | DM | cord | 1.78 | 1.78 | 1.56 | 513.33 | 446.00 | 483.78 |
| fear | GL | cord | 1.89 | 1.67 | 1.78 | 736.00 | 485.44 | 472.11 |
| fear | DM | fur | 1.78 | 1.78 | 1.33 | 357.33 | 321.22 | 533.44 |
| fear | GL | fur | 1.78 | 1.33 | 1.56 | 708.78 | 553.22 | 505.78 |
| fear | DM | hay | 1.89 | 1.78 | 1.89 | 425.44 | 901.56 | 454.00 |
| fear | GL | hay | 1.89 | 1.33 | 1.78 | 750.33 | 883.22 | 420.67 |
| fear | DM | jug | 1.67 | 2.11 | 1.11 | 444.67 | 680.44 | 334.78 |
| fear | GL | jug | 1.78 | 1.67 | 1.78 | 385.11 | 449.67 | 328.67 |
| fear | DM | lawn | 2.67 | 1.89 | 1.67 | 753.67 | 373.67 | 558.00 |
| fear | GL | lawn | 1.78 | 1.22 | 1.78 | 570.56 | 458.22 | 376.78 |
| fear | DM | spray | 1.78 | 2.11 | 1.78 | 386.56 | 424.89 | 403.22 |
| fear | GL | spray | 1.89 | 1.78 | 1.67 | 539.11 | 572.22 | 654.22 |
| fear | DM | truck | 1.56 | 2.22 | 1.89 | 581.11 | 446.33 | 517.78 |
| fear | GL | truck | 1.67 | 1.56 | 1.44 | 702.00 | 348.67 | 492.44 |
| fear | DM | vest | 1.78 | 1.89 | 1.78 | 491.33 | 456.89 | 571.56 |
| fear | GL | vest | 1.67 | 1.89 | 1.67 | 294.11 | 551.56 | 1094.22 |
|  |  | **MEAN** | **1.83** | **1.75** | **1.70** | **518.18** | **534.42** | **524.32** |

Note that disgust stimuli obtained high disgust intensity ratings whereas fear stimuli obtained low disgust intensity ratings across audio-visual and corresponding unimodal auditory and unimodal visual stimuli for each word. Also note that mean rating RTs for audio-visual stimuli were faster than were mean rating RTs for both unimodal auditory and unimodal visual stimuli.

**Table S3** shows the mean fear intensity ratings given by participants for the selected fear and disgust stimuli.

**Table S3.** Mean fear intensity rating responses and response times for stimuli selected from actors DM and GL.

|  |  |  | **Mean Fear Rating** | | | **Mean Rating RT** | | |
| --- | --- | --- | --- | --- | --- | --- | --- | --- |
|  |  |  | **(1=not at all fearful 7=very fearful)** | | | **(in ms)** | | |
| **Stimulus Type** | **Actor** | **Word** | **Audio-visual** | **Auditory** | **Visual** | **Audio-visual** | **Auditory** | **Visual** |
| fear | DM | bowl | 6.89 | 5.44 | 5.78 | 320.67 | 557.44 | 718.22 |
| fear | GL | bowl | 5.89 | 6.22 | 6.00 | 666.11 | 573.89 | 505.22 |
| fear | DM | chair | 7.00 | 6.11 | 6.56 | 323.56 | 451.11 | 254.44 |
| fear | GL | chair | 6.22 | 6.44 | 6.78 | 504.56 | 634.00 | 237.33 |
| fear | DM | cord | 6.78 | 6.67 | 6.11 | 364.44 | 296.56 | 646.56 |
| fear | GL | cord | 6.56 | 5.78 | 6.89 | 430.00 | 387.33 | 248.67 |
| fear | DM | fur | 6.78 | 6.11 | 6.33 | 221.89 | 430.11 | 472.56 |
| fear | GL | fur | 5.44 | 4.11 | 6.44 | 421.00 | 683.33 | 596.89 |
| fear | DM | hay | 6.56 | 6.00 | 6.44 | 514.22 | 536.22 | 436.11 |
| fear | GL | hay | 5.78 | 4.00 | 5.22 | 841.11 | 899.67 | 381.11 |
| fear | DM | jug | 6.78 | 6.78 | 5.44 | 385.78 | 597.44 | 398.11 |
| fear | GL | jug | 6.67 | 6.22 | 6.44 | 422.89 | 668.00 | 362.00 |
| fear | DM | lawn | 7.00 | 6.22 | 6.22 | 190.22 | 972.78 | 398.67 |
| fear | GL | lawn | 6.67 | 6.00 | 6.78 | 338.00 | 511.56 | 473.11 |
| fear | DM | spray | 6.22 | 6.33 | 7.00 | 349.00 | 532.33 | 385.33 |
| fear | GL | spray | 6.78 | 5.11 | 6.78 | 377.56 | 916.89 | 349.78 |
| fear | DM | truck | 7.00 | 6.67 | 6.56 | 374.44 | 319.89 | 422.44 |
| fear | GL | truck | 6.78 | 5.44 | 7.00 | 329.44 | 325.22 | 210.89 |
|  |  | **MEAN** | **6.54** | **5.87** | **6.38** | **409.72** | **571.88** | **416.52** |
|  |  |  |  |  |  |  |  |  |
| disgust | DM | bowl | 1.78 | 1.67 | 1.78 | 355.67 | 654.33 | 346.22 |
| disgust | GL | bowl | 1.89 | 1.89 | 1.33 | 1001.56 | 661.33 | 370.89 |
| disgust | DM | chair | 1.11 | 1.33 | 1.56 | 279.78 | 489.11 | 322.78 |
| disgust | GL | chair | 1.22 | 1.78 | 1.78 | 289.11 | 597.89 | 509.78 |
| disgust | DM | cord | 1.44 | 1.33 | 1.67 | 505.78 | 690.78 | 541.44 |
| disgust | GL | cord | 2.22 | 1.44 | 2.00 | 639.00 | 614.56 | 298.78 |
| disgust | DM | fur | 1.11 | 2.22 | 1.78 | 322.00 | 989.11 | 284.89 |
| disgust | GL | fur | 1.22 | 2.11 | 1.33 | 479.33 | 851.22 | 546.33 |
| disgust | DM | hay | 1.78 | 1.67 | 1.67 | 489.89 | 595.00 | 271.33 |
| disgust | GL | hay | 1.56 | 1.78 | 2.33 | 386.56 | 638.44 | 392.78 |
| disgust | DM | jug | 1.11 | 1.33 | 1.44 | 480.44 | 565.22 | 381.44 |
| disgust | GL | jug | 1.89 | 1.11 | 1.00 | 584.22 | 745.67 | 472.00 |
| disgust | DM | lawn | 1.22 | 1.78 | 1.22 | 288.78 | 449.11 | 311.11 |
| disgust | GL | lawn | 1.56 | 1.11 | 2.22 | 536.00 | 493.33 | 998.44 |
| disgust | DM | spray | 1.33 | 1.44 | 1.11 | 397.33 | 494.44 | 434.67 |
| disgust | GL | spray | 1.78 | 1.56 | 1.89 | 390.11 | 397.00 | 700.78 |
| disgust | DM | truck | 1.11 | 1.33 | 1.11 | 488.00 | 467.00 | 387.67 |
| disgust | GL | truck | 1.78 | 2.56 | 1.44 | 309.67 | 735.00 | 402.67 |
|  |  | **MEAN** | **1.51** | **1.64** | **1.59** | **456.85** | **618.25** | **443.00** |

Note that fear stimuli obtained high fear intensity ratings whereas disgust stimuli obtained low fear intensity ratings across audio-visual and corresponding unimodal auditory and unimodal visual stimuli for each word. Also note that, on average, participants took longer to rate unimodal auditory stimuli than audio-visual or unimodal visual stimuli (see mean rating RTs in **Table S2 & Table S3**).

Using the disgust and fear intensity ratings for each stimulus, it was possible to ensure that each stimulus was highly distinguishable from the unintended emotion. **Table S4** shows the difference in mean ratings for both fear and disgust stimuli.

**Table S4.** Mean intensity rating differences. For each stimulus, the difference between disgust and fear mean intensity ratings and the difference between fear and disgust mean intensity ratings are presented.

|  |  | **Mean Rating Difference:** | | | **Mean Rating Difference:** | | |
| --- | --- | --- | --- | --- | --- | --- | --- |
|  |  | **Disgust Minus Fear** | | | **Fear Minus Disgust** | | |
| **Actor** | **Word** | **Audio-visual** | **Auditory** | **Visual** | **Audio-visual** | **Auditory** | **Visual** |
| DM | bowl | 6.56 | 5.11 | 6.67 | 5.11 | 3.77 | 4.00 |
| GL | bowl | 6.44 | 5.78 | 5.89 | 4.00 | 4.33 | 4.67 |
| DM | chair | 6.44 | 4.56 | 6.00 | 5.89 | 4.78 | 5.00 |
| GL | chair | 6.56 | 5.78 | 6.78 | 5.00 | 4.66 | 5.00 |
| DM | cord | 6.78 | 4.44 | 6.67 | 5.34 | 5.34 | 4.44 |
| GL | cord | 6.22 | 5.11 | 5.67 | 4.34 | 4.34 | 4.89 |
| DM | fur | 6.78 | 5.67 | 6.56 | 5.67 | 3.89 | 4.55 |
| GL | fur | 6.67 | 5.11 | 6.33 | 4.22 | 2.00 | 5.11 |
| DM | hay | 6.67 | 4.00 | 6.44 | 4.78 | 4.33 | 4.77 |
| GL | hay | 6.67 | 4.33 | 6.67 | 4.22 | 2.22 | 2.89 |
| DM | jug | 6.67 | 4.67 | 6.56 | 5.67 | 5.45 | 4.00 |
| GL | jug | 6.00 | 4.11 | 5.89 | 4.78 | 5.11 | 5.44 |
| DM | lawn | 6.33 | 4.22 | 6.22 | 5.78 | 4.44 | 5.00 |
| GL | lawn | 6.44 | 4.44 | 6.44 | 5.11 | 4.89 | 4.56 |
| DM | spray | 6.56 | 5.56 | 6.78 | 4.89 | 4.89 | 5.89 |
| GL | spray | 6.22 | 5.56 | 6.33 | 5.00 | 3.55 | 4.89 |
| DM | truck | 6.00 | 6.00 | 6.33 | 5.89 | 5.34 | 5.45 |
| GL | truck | 6.56 | 5.22 | 6.56 | 5.00 | 2.88 | 5.56 |
| DM | vest | 6.33 | 4.44 | 6.56 | 5.33 | 5.12 | 5.00 |
| GL | vest | 6.00 | 4.22 | 6.22 | 5.67 | 4.77 | 5.34 |
|  | **MEAN** | **6.45** | **4.92** | **6.38** | **5.08** | **4.31** | **4.82** |

The second criterion for stimulus selection sought to eliminate the inclusion of emotionally confusable fear and disgust stimuli by requiring that each stimulus be distinguishable from the unintended emotion. If the fear and disgust ratings for a given stimulus were discrepant by a factor of 2 or more, then the stimulus was considered as highly distinguishable from the unintended emotion. As shown in **Table S4**, every audio-visual, unimodal auditory and unimodal visual stimulus met this secondary criterion.

When considered together, **Tables S2** and **S3** show that the stimuli selected from actors DM and GL were highly recognizable as the intended emotion and therefore met the first criterion detailed above. Furthermore, **Table S4** shows that audio-visual and corresponding unimodal auditory and unimodal visual stimuli met the secondary criterion of high distinction from the unintended emotion.

**Table S5.** Coordinates in MNI space and associated peak t-scores showing the significant differences (one-tailed) in power for the main effect of congruent audio-visual emotion minus (auditory emotion + visual emotion). Positive t-scores reflect significant increases in power whereas negative t-values reflect significant decreases in power.

| ***Brain Regions*** |  |  |  | **Coordinates** | | |
| --- | --- | --- | --- | --- | --- | --- |
|  | **BA** | **P Value** | **T Score** | **X** | **Y** | **Z** |
| ***Theta (4-8Hz)*** |  |  |  |  |  |  |
| ***AV Congruent– (A + V)*** |  |  |  |  |  |  |
| **0-500ms** |  |  |  |  |  |  |
| R Superior Temporal gyrus, R Superior Temporal sulcus, R Middle Temporal gyrus | 21 | <.005 | -2.94 | 64 | -10 | -2 |
| R Thalamus, R Hippocampus, R Putamen | - | <.010 | -2.72 | 10 | -30 | 4 |
| R Thalamus, R Hippocampus, R Putamen | - | <.010 | -2.70 | 10 | -20 | 8 |
| R Hippocampus, R Putamen, R Thalamus | - | <.010 | -2.60 | 24 | -36 | 8 |
| R Middle Temporal gyrus, R Superior Temporal sulcus, R Superior Temporal gyrus | 21 | <.015 | -2.54 | 70 | -30 | -6 |
|  |  |  |  |  |  |  |
| ***Theta (4-8Hz)*** |  |  |  |  |  |  |
| ***AV Congruent – (A + V)*** |  |  |  |  |  |  |
| **50-550ms** |  |  |  |  |  |  |
| R Superior Temporal gyrus, R Superior Temporal sulcus, R Middle Temporal gyrus | 21 | <.010 | -2.74 | 70 | -6 | -2 |
| R Thalamus, R Hippocampus, L Thalamus, L Posterior Cingulate cortex | - | <.010 | -2.73 | 4 | -26 | -2 |
| R Thalamus, R Caudate, R Hippocampus, L Thalamus, L Posterior Cingulate cortex | - | <.015 | -2.50 | 14 | -10 | 18 |
| L Thalamus, L Posterior Cingulate cortex, R Hippocampus, R Caudate, R Thalamus | - | <.015 | -2.42 | -6 | -20 | 18 |
| L Inferior Frontal gyrus | 45 | <.020 | -2.31 | -56 | 24 | 8 |
|  |  |  |  |  |  |  |
| ***Theta (4-8Hz)*** |  |  |  |  |  |  |
| ***AV Congruent– (A + V)*** |  |  |  |  |  |  |
| **100-600ms** |  |  |  |  |  |  |
| R Thalamus, R Caudate, R Putamen, R Anterior Cingulate cortex | - | <.005 | -3.01 | 4 | -26 | -2 |
| R Caudate,R Putamen, R Thalamus, R Anterior Cingulate cortex | - | <.005 | -2.95 | 10 | 0 | 18 |
| R Anterior Cingulate cortex, R Thalamus, R Caudate, R Putamen | 33 | <.005 | -2.94 | 4 | 14 | 28 |
| R Thalamus, R Caudate, R Putamen, R Anterior Cingulate cortex | - | <.010 | -2.85 | 10 | -20 | 8 |
| R Thalamus, R Caudate, R Putamen, R Anterior Cingulate cortex | - | <.010 | -2.84 | 14 | -10 | 14 |
| L Lateral Frontal Pole | 10 | <.030 | -2.05 | -44 | 50 | 0 |
| R Superior Temporal gyrus | 22 | <.035 | -1.99 | 70 | -6 | 0 |
|  |  |  |  |  |  |  |
| ***Theta (4-8Hz)*** |  |  |  |  |  |  |
| ***AV Congruent – (A + V)*** |  |  |  |  |  |  |
| **150-650ms** |  |  |  |  |  |  |
| R Thalamus, R Caudate, R Hippocampus, L Thalamus, L Posterior Cingulate cortex | - | <.005 | -3.13 | 10 | -6 | 14 |
| L Thalamus, L Posterior Cingulate cortex, R Caudate, R Hippocampus, R Thalamus | - | <.005 | -3.00 | -6 | -26 | 4 |
| L Thalamus, L Posterior Cingulate cortex, R Caudate, R Hippocampus, R Thalamus | - | <.005 | -2.98 | -6 | -20 | 14 |
| L Lateral Frontal Pole | 10 | <.010 | -2.64 | -50 | 50 | 4 |
| L Lateral Frontal Pole | 47 | <.015 | -2.42 | -40 | 44 | -6 |
|  |  |  |  |  |  |  |
| ***Theta (4-8Hz)*** |  |  |  |  |  |  |
| ***AV Congruent – (A + V)*** |  |  |  |  |  |  |
| **200-700ms** |  |  |  |  |  |  |
| R Precuneus | 7 | <.025 | 2.20 | 4 | -70 | 68 |
| L Thalamus, L Precentral gyrus, R Thalamus, R Caudate, R Anterior Cingulate cortex, R Precentral gyrus | - | <.005 | -3.03 | 0 | -20 | 8 |
| L Lateral Frontal Pole | 46 | <.010 | -2.64 | -50 | 44 | 8 |
| L Precentral gyrus, L Thalamus, R Thalamus, R Caudate, R Anterior Cingulate cortex, R Precentral gyrus | 6 | <.015 | -2.48 | -26 | -16 | 34 |
| R Precentral gyrus, R Postcentral gyrus | 6 | <.015 | -2.42 | 64 | 10 | 28 |
| R Superior Frontal gyrus, R Anterior Cingulate cortex, R Caudate, R Thalamus, L Thalamus, L Precentral gyrus, R Precentral gyrus, R Postcentral gyrus | 8 | <.015 | -2.41 | 4 | 24 | 54 |
|  |  |  |  |  |  |  |
| ***Theta (4-8Hz)*** |  |  |  |  |  |  |
| ***AV Congruent– (A + V)*** |  |  |  |  |  |  |
| **250-750ms** |  |  |  |  |  |  |
| R Postcentral gyrus, R Superior Parietal loule | 5 | <.020 | 2.35 | 4 | -56 | 62 |
| R Superior Parietal lobule, R Postcentral gyrus | 7 | <.025 | 2.17 | 0 | -56 | 64 |
| L Lateral Frontal Pole | 11 | <.010 | -2.67 | -36 | 50 | -16 |
| R Thalamus, L Thalamus | - | <.020 | -2.34 | 4 | -16 | 8 |
| R Precentral gyrus | 6 | <.025 | -2.11 | 64 | 10 | 28 |
| R Superior Frontal gyrus | 8 | <.030 | -2.02 | 4 | 24 | 54 |
|  |  |  |  |  |  |  |
| ***Theta (4-8Hz)*** |  |  |  |  |  |  |
| ***AV Congruent– (A + V)*** |  |  |  |  |  |  |
| **300-800ms** |  |  |  |  |  |  |
| L Lateral Frontal Pole | 11 | <.015 | -2.51 | -46 | 54 | -12 |
| L Lateral Frontal Pole | 11 | <.020 | -2.32 | -30 | 64 | -12 |
| R Thalamus, L Thalamus | - | <.020 | -2.31 | 4 | -10 | 14 |
| R Precentral gyrus | 6 | <.025 | -2.17 | 64 | 10 | 28 |
|  |  |  |  |  |  |  |
| **BA=Brodmann Area; L=Left; R=Right; n.s.=No significant areas** |  |  |  |  |  |  |

***Table S6.*** *Coordinates in MNI space and associated peak t-scores showing the significant differences (one-tailed) in power for the main effect of congruent audio-visual emotion minus (auditory emotion + visual emotion). Positive t-scores reflect significant increases in power whereas negative t-values reflect significant decreases in power.*

| ***Brain Regions*** |  |  |  | **Coordinates** | | |
| --- | --- | --- | --- | --- | --- | --- |
|  | **BA** | **P Value** | **T Score** | **X** | **Y** | **Z** |
| ***Alpha (8-13Hz)*** |  |  |  |  |  |  |
| ***AV Congruent– (A + V)*** |  |  |  |  |  |  |
| **0-500ms** |  |  |  |  |  |  |
| R Middle Temporal gyrus | 21 | <.015 | 2.41 | 64 | 0 | 22 |
| R mid-posterior Superior Temporal gyrus, R Superior Temporal sulcus | 22 | <.020 | 2.25 | -46 | -30 | -2 |
| R Precentral gyrus | 6 | <.035 | 1.96 | 40 | -10 | 38 |
| R Temporal Pole, R Anterior Superior Temporal gyrus | 38 | <.040 | 1.88 | 60 | 14 | -16 |
|  |  |  |  |  |  |  |
| ***Alpha (8-13Hz)*** |  |  |  |  |  |  |
| ***AV Congruent – (A + V)*** |  |  |  |  |  |  |
| **50-550ms** |  |  |  |  |  |  |
| R Superior Temporal gyrus, R Superior Temporal sulcus, R Temporal Pole | 38 | <.005 | 2.99 | 60 | 4 | -12 |
| R Precentral gyrus, R Postcentral gyrus, R Insula | 6 | <.005 | 2.80 | 40 | -6 | 34 |
| L Anterior Insula | 13 | <.040 | 1.90 | -30 | 20 | -2 |
|  |  |  |  |  |  |  |
| ***Alpha (8-13Hz)*** |  |  |  |  |  |  |
| ***AV Congruent– (A + V)*** |  |  |  |  |  |  |
| **100-600ms** |  |  |  |  |  |  |
| R Precentral gyrus, R Postcentral gyrus | 6 | <.005 | 3.13 | 34 | -6 | 34 |
| R Temporal pole, R Superior Temporal gyrus | 38 | <.010 | 2.87 | 60 | 10 | -12 |
| R Superior Temporal gyrus, R Temporal Pole | 22 | <.015 | 2.45 | 64 | 0 | -6 |
| L Insula | 47 | <.025 | 2.15 | -30 | 14 | -2 |
| R Superior Temporal gyrus, R Superior Temporal sulcus | 21 | <.030 | 2.09 | 70 | -16 | -2 |
|  |  |  |  |  |  |  |
| ***Alpha (8-13Hz)*** |  |  |  |  |  |  |
| ***AV Congruent – (A + V)*** |  |  |  |  |  |  |
| **150-650ms** |  |  |  |  |  |  |
| R Precentral gyrus, R Postcetntral gyrus | 6 | <.005 | 3.15 | 34 | -6 | 28 |
| R Planum Temporale, R Superior Temporal gyrus, R Postcentral gyrus, R Precentral gyrus | 42 | <.025 | 2.19 | 64 | -16 | 8 |
| L Insula | 47 | <.025 | 2.13 | -30 | 14 | -2 |
| R Supramarginal gyrus | 40 | <.035 | 1.96 | 60 | -40 | 44 |
| R Temporal Pole | 22 | <.040 | 1.90 | 54 | 10 | -6 |
| R Medial Frontal Pole | 11 | <.030 | -2.10 | 4 | 70 | -12 |
|  |  |  |  |  |  |  |
| ***Alpha (8-13Hz)*** |  |  |  |  |  |  |
| ***AV Congruent – (A + V)*** |  |  |  |  |  |  |
| **200-700ms** |  |  |  |  |  |  |
| R Postcentral gyrus, R Precentral gyrus | 3 | <.005 | 3.37 | 34 | -10 | 34 |
| R Planum Temporale, R Postecentral gyrus | 43 | <.015 | 2.42 | 64 | -16 | 14 |
| R Supramarginal gyrus | 40 | <.025 | 2.14 | 54 | -40 | 44 |
| L Anterior Insula | 13 | <.050 | 1.78 | -36 | 20 | -6 |
| R Medial Frontal Pole | 11 | <.030 | -2.03 | 4 | 70 | -12 |
|  |  |  |  |  |  |  |
| ***Alpha (8-13Hz)*** |  |  |  |  |  |  |
| ***AV Congruent– (A + V)*** |  |  |  |  |  |  |
| **250-750ms** |  |  |  |  |  |  |
| R Precentral gyrus | 6 | <.020 | 2.34 | 34 | -6 | 38 |
| R Supramarginal gyrus | 40 | <.045 | 1.82 | 54 | -40 | 44 |
| R Medial Frontal Pole | 10 | <.020 | -2.22 | 4 | 70 | -6 |
| R Anterior Cingulate cortex | 32 | <.045 | -1.83 | 10 | 44 | 8 |
|  |  |  |  |  |  |  |
| ***Alpha (8-13Hz)*** |  |  |  |  |  |  |
| ***AV Congruent– (A + V)*** |  |  |  |  |  |  |
| **300-800ms** |  |  |  |  |  |  |
| R Precentral gyrus | 6 | <.020 | 2.28 | 34 | -6 | 38 |
| R Angular gyrus | 40 | <.050 | 1.76 | 44 | -56 | 58 |
| R Medial Frontal Pole | 10 | <.030 | -2.10 | 4 | 70 | -6 |
| R Lateral Frontal Pole | 11 | <.035 | -1.99 | 44 | 40 | -16 |
| R Anterior Cingulate cortex | 32 | <.045 | -1.86 | 10 | 40 | 4 |
| R Anterior Cingulate cortex | 24 | <.045 | -1.86 | 10 | 30 | 14 |
|  |  |  |  |  |  |  |
| **BA=Brodmann Area; L=Left; R=Right; n.s.=No significant areas** |  |  |  |  |  |  |

***Table S7.*** *Coordinates in MNI space and associated peak t-scores showing the significant differences (one-tailed) in power for the main effect of congruent audio-visual emotion minus (auditory emotion + visual emotion). Positive t-scores reflect significant increases in power whereas negative t-values reflect significant decreases in power.*

| ***Brain Regions*** |  |  |  | **Coordinates** | | |
| --- | --- | --- | --- | --- | --- | --- |
|  | **BA** | **P Value** | **T Score** | **X** | **Y** | **Z** |
| ***Gamma (30-80Hz)*** |  |  |  |  |  |  |
| ***AV Congruent– (A + V)*** |  |  |  |  |  |  |
| **0-500ms** |  |  |  |  |  |  |
| n.s. |  |  |  |  |  |  |
|  |  |  |  |  |  |  |
| ***Gamma (30-80Hz)*** |  |  |  |  |  |  |
| ***AV Congruent – (A + V)*** |  |  |  |  |  |  |
| **50-550ms** |  |  |  |  |  |  |
| R Superior Temporal gyrus, R Planum Temporale, R Postcentral gyrus | 22 | <.010 | 2.77 | 70 | -10 | 4 |
|  |  |  |  |  |  |  |
| ***Gamma (30-80Hz)*** |  |  |  |  |  |  |
| ***AV Congruent– (A + V)*** |  |  |  |  |  |  |
| **100-600ms** |  |  |  |  |  |  |
| R Superior Temporal gyrus, R Planum Temporale, R Postcentral gyrus | 21 | <.015 | 2.48 | 70 | -10 | -2 |
| R Postcentral gyrus | 3 | <.030 | 2.04 | 64 | -16 | 24 |
|  |  |  |  |  |  |  |
| ***Gamma (30-80Hz)*** |  |  |  |  |  |  |
| ***AV Congruent – (A + V)*** |  |  |  |  |  |  |
| **150-650ms** |  |  |  |  |  |  |
| R Superior Temporal gyrus, R Planum Temporale | 22 | <.025 | 2.18 | 70 | -10 | 4 |
|  |  |  |  |  |  |  |
| ***Gamma (30-80Hz)*** |  |  |  |  |  |  |
| ***AV Congruent – (A + V)*** |  |  |  |  |  |  |
| **200-700ms** |  |  |  |  |  |  |
| R Superior Temporal gyrus, R Planum Temporale | 21 | <.005 | 2.94 | 70 | -10 | -2 |
|  |  |  |  |  |  |  |
| ***Gamma (30-80Hz)*** |  |  |  |  |  |  |
| ***AV Congruent– (A + V)*** |  |  |  |  |  |  |
| **250-750ms** |  |  |  |  |  |  |
| n.s. |  |  |  |  |  |  |
|  |  |  |  |  |  |  |
| ***Gamma (30-80Hz)*** |  |  |  |  |  |  |
| ***AV Congruent– (A + V)*** |  |  |  |  |  |  |
| **300-800ms** |  |  |  |  |  |  |
| R Postcentral gyrus | 43 | <.035 | 2.00 | 60 | -10 | 18 |
|  |  |  |  |  |  |  |
| **BA=Brodmann Area; L=Left; R=Right; n.s.=No significant areas** |  |  |  |  |  |  |

**Table S8.** Coordinates in MNI space and associated peak t-scores showing the significant differences (one-tailed) in power for the main effect of incongruent audio-visual emotion minus (auditory emotion + visual emotion). Positive t-scores reflect significant increases in power whereas negative t-values reflect significant decreases in power.

| ***Brain Regions*** |  |  |  | **Coordinates** | | |
| --- | --- | --- | --- | --- | --- | --- |
|  | **BA** | **P Value** | **T Score** | **X** | **Y** | **Z** |
| ***Theta (4-8Hz)*** |  |  |  |  |  |  |
| ***AV Incongruent– (A + V)*** |  |  |  |  |  |  |
| **0-500ms** |  |  |  |  |  |  |
| L Middle Frontal gyrus | 9 | <.015 | 2.54 | -30 | 34 | 34 |
| R Cerebellum | - | <.030 | 2.05 | 50 | -46 | -56 |
| R Cerebellum | - | <.040 | 1.86 | 34 | -40 | -42 |
|  |  |  |  |  |  |  |
| ***Theta (4-8Hz)*** |  |  |  |  |  |  |
| ***AV Incongruent – (A + V)*** |  |  |  |  |  |  |
| **50-550ms** |  |  |  |  |  |  |
| L Middle Frontal gyrus | 9 | <.030 | 2.03 | -30 | 34 | 34 |
| R Cerebellum | - | <.045 | 1.85 | 50 | -46 | -56 |
| R Anterior Superior Temporal gyrus, | 21 | <.045 | -1.85 | 70 | -6 | -2 |
| L Lateral Frontal Pole | 10 | <.045 | -1.84 | -46 | 50 | 14 |
|  |  |  |  |  |  |  |
| ***Theta (4-8Hz)*** |  |  |  |  |  |  |
| ***AV Incongruent– (A + V)*** |  |  |  |  |  |  |
| **100-600ms** |  |  |  |  |  |  |
| L Superior Temporal gyrus | 42 | <.015 | -2.42 | -66 | -26 | 8 |
| R Superior Frontal gyrus | 6 | <.025 | -2.14 | 14 | 14 | 54 |
| L Lateral Frontal Pole | 10 | <.030 | -2.07 | -40 | 50 | 8 |
| R Superior Frontal gyrus | 8 | <.030 | -2.02 | 24 | 34 | 44 |
| R Thalamus, R Caudate | - | <.035 | -1.93 | 10 | -20 | 8 |
|  |  |  |  |  |  |  |
| ***Theta (4-8Hz)*** |  |  |  |  |  |  |
| ***AV Incongruent – (A + V)*** |  |  |  |  |  |  |
| **150-650ms** |  |  |  |  |  |  |
| R Middle Temporal gyrus | 21 | <.020 | 2.22 | 60 | -50 | -2 |
| L Postcentral gyrus, L Heschl’s gyrus, | 40 | <.030 | -2.01 | -50 | -20 | 18 |
|  |  |  |  |  |  |  |
| ***Theta (4-8Hz)*** |  |  |  |  |  |  |
| ***AV Incongruent – (A + V)*** |  |  |  |  |  |  |
| **200-700ms** |  |  |  |  |  |  |
| R Middle Temporal gyrus | 21 | <.001 | 3.37 | 60 | -46 | -2 |
| L Middle Frontal gyrus | 10 | <.05 | 1.77 | -26 | 30 | 24 |
| R Superior Frontal gyrus | 6 | <.005 | -3.28 | 14 | 20 | 68 |
| R Precentral gyrus | 6 | <.015 | -2.40 | 64 | 10 | 28 |
| L Supramarginal gyrus | 40 | <.025 | -2.12 | -56 | -26 | 28 |
| L Supramarginal gyrus | 40 | <.035 | -1.94 | -66 | -50 | 24 |
| R Fontal Pole | 8 | <.035 | -1.90 | 30 | 40 | 48 |
|  |  |  |  |  |  |  |
| ***Theta (4-8Hz)*** |  |  |  |  |  |  |
| ***AV Incongruent– (A + V)*** |  |  |  |  |  |  |
| **250-750ms** |  |  |  |  |  |  |
| R Middle Temporal gyrus, R Inferior Temporal gyrus, R Superior Temporal sulcus, R Posterior Superior Temporal gyrus | 21 | <.001 | 3.62 | 64 | -40 | -2 |
| L Middle Frontal gyrus, L Anterior Cingulate cortex | 46 | <.010 | 2.56 | -26 | 24 | 24 |
| R Middle Temporal gyrus, R Inferior Temporal gyrus, R Superior Temporal sulcus, R Posterior Superior Temporal gyrus | 21 | <.020 | 2.31 | 70 | -20 | -22 |
| L Superior Temporal gyrus | 21 | <.025 | 2.20 | -66 | -16 | -2 |
| R Hippocampus | - | <.035 | 1.93 | 30 | -40 | 4 |
|  |  |  |  |  |  |  |
| ***Theta (4-8Hz)*** |  |  |  |  |  |  |
| ***AV Incongruent– (A + V)*** |  |  |  |  |  |  |
| **300-800ms** |  |  |  |  |  |  |
| R Supramarginal gyrus, R Middle Temporal gyrus, R Inferior Temporal gyrus | 22 | <.005 | 3.52 | 60 | -40 | 8 |
| R Middle Temporal gyrus, R Supramarginal gyrus, R Inferior Temporal gyrus | 21 | <.005 | 3.32 | 70 | -40 | 4 |
| R Middle Temporal gyrus, R Supramarginal gyrus, R Inferior Temporal gyrus | 21 | <.005 | 2.97 | 70 | -20 | -22 |
| L Middle Frontal gyrus, L Inferior Frontal gyrus, L Superior Frontal gyrus, L Anterior Cingulate cortex, L Mid-Cingulate cortex, L Caudate | 10 | <.005 | 2.90 | -30 | 30 | 28 |
| L Superior Temporal gyrus, L Postcentral gyrus, L Temporal Pole | 22 | <.010 | 2.78 | -66 | -16 | 4 |
|  |  |  |  |  |  |  |
| **BA=Brodmann Area; L=Left; R=Right; n.s.=No significant areas** |  |  |  |  |  |  |

**Table S9.** Coordinates in MNI space and associated peak t-scores showing the significant differences (one-tailed) in power for the main effect of incongruent audio-visual emotion minus (auditory emotion + visual emotion). Positive t-scores reflect significant increases in power whereas negative t-values reflect significant decreases in power.

| ***Brain Regions*** |  |  |  | **Coordinates** | | |
| --- | --- | --- | --- | --- | --- | --- |
|  | **BA** | **P Value** | **T Score** | **X** | **Y** | **Z** |
| ***Alpha (8-13Hz)*** |  |  |  |  |  |  |
| ***AV Incongruent– (A + V)*** |  |  |  |  |  |  |
| **0-500ms** |  |  |  |  |  |  |
| R Postcentral gyrus, R Precentral gyrus | 2 | <.020 | 2.35 | 34 | -16 | 34 |
| R Posterior Superior Temporal gyrus | 22 | <.045 | 1.86 | 54 | -30 | 4 |
|  |  |  |  |  |  |  |
| ***Alpha (8-13Hz)*** |  |  |  |  |  |  |
| ***AV Incongruent – (A + V)*** |  |  |  |  |  |  |
| **50-550ms** |  |  |  |  |  |  |
| R Precentral gyrus, R Postcentral gyrus, R Planum Temporale,  R Heschl's gyrus | 6 | <.020 | 2.31 | 34 | -16 | 34 |

| R Mid-Posterior Superior Temporal gyrus, R Planum Temporale | 42 | <.035 | 1.95 | 60 | -26 | 8 |
| --- | --- | --- | --- | --- | --- | --- |
| R Middle Frontal gyrus, R Caudate | 46 | <.030 | -2.09 | 24 | 24 | 18 |
|  |  |  |  |  |  |  |
| ***Alpha (8-13Hz)*** |  |  |  |  |  |  |
| ***AV Incongruent– (A + V)*** |  |  |  |  |  |  |
| **100-600ms** |  |  |  |  |  |  |
| R Planum Temporale | 41 | <.050 | 1.75 | 60 | -20 | 8 |
| R Middle Frontal gyrus, R caudate | 46 | <.015 | -2.55 | 40 | 20 | 24 |
| R Caudate, R Insula, R Inferior Frontal gyrus | - | <.015 | -2.44 | 20 | 14 | 24 |
| R Medial Frontal Pole | 11 | <.020 | -2.26 | 10 | 70 | -12 |
|  |  |  |  |  |  |  |
| ***Alpha (8-13Hz)*** |  |  |  |  |  |  |
| ***AV Incongruent – (A + V)*** |  |  |  |  |  |  |
| **150-650ms** |  |  |  |  |  |  |
| R Postcentral gyrus | 43 | <.050 | 1.76 | 54 | -16 | 18 |
| R Medial Frontal Pole | 11 | <.010 | -2.59 | 4 | 70 | -12 |
| R Inferior Frontal gyrus | 45 | <.030 | -2.07 | 40 | 20 | 14 |
| R Caudate | - | <.040 | -1.88 | 24 | 10 | 18 |
| R Middle Frontal gyrus | 46 | <.045 | -1.85 | 50 | 24 | 24 |
|  |  |  |  |  |  |  |
| ***Alpha (8-13Hz)*** |  |  |  |  |  |  |
| ***AV Incongruent – (A + V)*** |  |  |  |  |  |  |
| **200-700ms** |  |  |  |  |  |  |
| R Postcentral gyrus | 43 | <.025 | 2.19 | 64 | -16 | 14 |
| R Postcentral gyrus | 2 | <.045 | 1.82 | 40 | -16 | 34 |
| R Postcentral gyrus | 2 | <.045 | 1.81 | 30 | -16 | 34 |
| R Medial Frontal Pole | 11 | <.010 | -2.57 | 10 | 70 | -12 |
| R Anterior Cingulate cortex | 33 | <.035 | -1.97 | 10 | 10 | 24 |
| R Inferior Frontal gyrus | 46 | <.040 | -1.87 | 40 | 20 | 18 |
| R Anterior Insula | 13 | <.045 | -1.84 | 30 | 14 | 18 |
|  |  |  |  |  |  |  |
| ***Alpha (8-13Hz)*** |  |  |  |  |  |  |
| ***AV Incongruent– (A + V)*** |  |  |  |  |  |  |
| **250-750ms** |  |  |  |  |  |  |
| R Postcentral gyrus | 42 | <.030 | 2.04 | 70 | -10 | 14 |
| R Medial Frontal Pole | 10 | <.010 | -2.58 | 4 | 70 | -6 |
| R Inferior Frontal gyrus, R Insula, R Putamen | 45 | <.010 | -2.53 | 34 | 20 | 18 |
| R Anterior Cingulate cortex, R Caudate | 33 | <.020 | -2.34 | 10 | 10 | 24 |
| R Anterior Insula, R Inferior Frontal gyrus | 13 | <.020 | -2.34 | 24 | 14 | 18 |
| R Inferior Frontal gyrus | 46 | <.045 | -1.77 | 54 | 34 | 14 |
|  |  |  |  |  |  |  |
| ***Alpha (8-13Hz)*** |  |  |  |  |  |  |
| ***AV Incongruent– (A + V)*** |  |  |  |  |  |  |
| **300-800ms** |  |  |  |  |  |  |
| R Medial Frontal Pole | 10 | <.015 | -2.47 | 4 | 70 | -6 |
| R Caudate, R Anterior Cingulate cortex, R Insula, R Inferior Frontal gyrus | - | <.015 | -2.45 | 20 | 20 | 14 |
| R Anterior Cingulate cortex, R Caudate, R Insula, R Inferior Frontal gyrus | 33 | <.015 | -2.42 | 10 | 14 | 24 |
| R Lateral Frontal Pole | 47 | <.050 | -1.79 | 50 | 44 | -12 |
|  |  |  |  |  |  |  |
| **BA=Brodmann Area; L=Left; R=Right; n.s.=No significant areas** |  |  |  |  |  |  |

**Table S10.** Coordinates in MNI space and associated peak t-scores showing the significant differences (one-tailed) in power for the main effect of incongruent audio-visual emotion minus (auditory emotion + visual emotion). Positive t-scores reflect significant increases in power whereas negative t-values reflect significant decreases in power.

| ***Brain Regions*** |  |  |  | **Coordinates** | | |
| --- | --- | --- | --- | --- | --- | --- |
|  | **BA** | **P Value** | **T Score** | **X** | **Y** | **Z** |
| ***Gamma (30-80Hz)*** |  |  |  |  |  |  |
| ***AV Incongruent– (A + V)*** |  |  |  |  |  |  |
| **0-500ms** |  |  |  |  |  |  |
| R Postcentral gyrus | 2 | <.045 | 1.81 | 64 | -26 | 48 |
|  |  |  |  |  |  |  |
| ***Gamma (30-80Hz)*** |  |  |  |  |  |  |
| ***AV Incongruent – (A + V)*** |  |  |  |  |  |  |
| **50-550ms** |  |  |  |  |  |  |
| n.s. |  |  |  |  |  |  |
|  |  |  |  |  |  |  |
| ***Gamma (30-80Hz)*** |  |  |  |  |  |  |
| ***AV Incongruent– (A + V)*** |  |  |  |  |  |  |
| **100-600ms** |  |  |  |  |  |  |
| n.s. |  |  |  |  |  |  |
|  |  |  |  |  |  |  |
| ***Gamma (30-80Hz)*** |  |  |  |  |  |  |
| ***AV Incongruent – (A + V)*** |  |  |  |  |  |  |
| **150-650ms** |  |  |  |  |  |  |
| n.s. |  |  |  |  |  |  |
|  |  |  |  |  |  |  |
| ***Gamma (30-80Hz)*** |  |  |  |  |  |  |
| ***AV Incongruent – (A + V)*** |  |  |  |  |  |  |
| **200-700ms** |  |  |  |  |  |  |
| R Superior Temporal gyrus | 21 | <.040 | 1.90 | 70 | -10 | -2 |
|  |  |  |  |  |  |  |
| ***Gamma (30-80Hz)*** |  |  |  |  |  |  |
| ***AV Incongruent– (A + V)*** |  |  |  |  |  |  |
| **250-750ms** |  |  |  |  |  |  |
| L Middle Temporal gyrus, L Superior Temporal sulcus, L Superior Temporal gyrus | 21 | <.035 | 1.99 | -66 | -30 | -2 |
|  |  |  |  |  |  |  |
| ***Gamma (30-80Hz)*** |  |  |  |  |  |  |
| ***AV Incongruent– (A + V)*** |  |  |  |  |  |  |
| **300-800ms** |  |  |  |  |  |  |
| L Middle Temporal gyrus, L Superior Temporal sulcus, L Superior Temporal gyrus, | 21 | <.010 | 2.73 | -66 | -40 | -6 |
| R Postcentral gyrus | 3 | <.045 | 1.83 | 70 | -10 | 28 |
|  |  |  |  |  |  |  |
| **BA=Brodmann Area; L=Left; R=Right; n.s.=No significant areas** |  |  |  |  |  |  |

**Supporting References**

1. Bradley MM, Lang PJ (1999) Affective norms for English words (ANEW): Stimuli, instruction manual and affective ratings. Technical report C-1, Gainesville, FL.: The Center for Research in Psychophysiology, University of Florida.

2. Kucera H, Francis WN (1967) Computational Analysis of Present-day American Engish. Providence: Brown Unviersity press.
